# The Chronotron: A Neuron That Learns to Fire Temporally Precise Spike Patterns

**DOI:** 10.1371/journal.pone.0040233

**Published:** 2012-08-06

**Authors:** Răzvan V. Florian

**Affiliations:** 1 Center for Cognitive and Neural Studies (Coneural), Romanian Institute of Science and Technology, Cluj-Napoca, Romania; University of Michigan, United States of America

## Abstract

In many cases, neurons process information carried by the precise timings of spikes. Here we show how neurons can learn to generate specific temporally precise output spikes in response to input patterns of spikes having precise timings, thus processing and memorizing information that is entirely temporally coded, both as input and as output. We introduce two new supervised learning rules for spiking neurons with temporal coding of information (chronotrons), one that provides high memory capacity (E-learning), and one that has a higher biological plausibility (I-learning). With I-learning, the neuron learns to fire the target spike trains through synaptic changes that are proportional to the synaptic currents at the timings of real and target output spikes. We study these learning rules in computer simulations where we train integrate-and-fire neurons. Both learning rules allow neurons to fire at the desired timings, with sub-millisecond precision. We show how chronotrons can learn to classify their inputs, by firing identical, temporally precise spike trains for different inputs belonging to the same class. When the input is noisy, the classification also leads to noise reduction. We compute lower bounds for the memory capacity of chronotrons and explore the influence of various parameters on chronotrons' performance. The chronotrons can model neurons that encode information in the time of the first spike relative to the onset of salient stimuli or neurons in oscillatory networks that encode information in the phases of spikes relative to the background oscillation. Our results show that firing one spike per cycle optimizes memory capacity in neurons encoding information in the phase of firing relative to a background rhythm.

## Introduction

There is increasing evidence that information is represented in the brain through the precise timing of spikes (temporally coded), not only through the neural firing rate [1]–[3]. For example, temporally structured multicell spiking patterns, organized into frames, were observed in hippocampus and cortex, and were associated to memory traces [4], [5]. In the olfactory bulb, spike latencies represent sensory input strength and identity [6], [7]. In the visual cortex, the relative spike timings of quasi-synchronized neurons, firing in sequences shorter than one cycle of beta/gamma oscillation, represent stimulus properties, and the information they carry grows with the oscillation strength [8]. The coding of information in the phases of spikes relative to a background oscillation has been observed in many brain regions, including the visual and prefrontal cortices and the hippocampus [9]–[14].

Learning in neural networks that represent information through a firing rate code has been thoroughly studied [15]; however, we have lacked efficient, theory-supported learning rules for spiking neurons with temporal coding of information. The tempotron, a model of a spiking neuron endowed with a specific learning rule, has shown how a neuron can give a binary response to information encoded in the precise timings of the afferent spikes [16]–[18]. But the tempotron's output represents information through the existence or the lack of an output spike during a predefined period. The timing of the tempotron's output spikes is arbitrary and does not carry information. Because of this change in the representation of information, a tempotron cannot be an information-carrying input for another tempotron. By contrast, the ReSuMe learning rule [19], [20] allows supervised learning of spiking neural codes where the output is also temporally coded, but this rule, as we will show, has a much lower memory capacity than the E-learning rule introduced here.

Here we present two new supervised learning rules for spiking neurons, which allow such neurons to process information that is encoded, for both input and output, in the precise timings of spikes. We show how single neurons can perform classification of input spike patterns into multiple categories, using a temporal coding of information with sub-millisecond precision. The E-learning rule that we introduce here is analytically derived, with approximations, and has a high memory capacity. The I-learning rule is heuristic, but is more biologically plausible, because synaptic changes depend directly on the synaptic currents at the timings (actual and target) of the postsynaptic spikes.

We first describe our results, by illustrating the chronotron problem, introducing our new learning rules, and describing their performance and their memory capacity. We then compare our results with previous ones. After a discussion of our results, the methods used for analytical derivations and computer simulations are presented in detail at the end of the paper.

## Results

### Understanding and illustrating the chronotron problem

We consider the problem of training a spiking neuron by changing its parameters, such that, for a given input, its output is as close as possible to some given target spike train (for how the target spike train may be provided in the brain, see the Discussion section). Multiple such input–output associations must be performed with a single set of neural parameters. Information is represented in both the input and the output through the precise timings of spikes. We call a neuron that solves this problem a chronotron.

In order to solve the chronotron problem, appropriate learning rules should be defined. Here we focus on learning rules that change the synaptic efficacies of the neuron, although other neural parameters can also be trained.

Our analysis uses the Spike Response Model (SRM) of spiking neurons, which reproduces with high accuracy the dynamics of the complex Hodgkin-Huxley neural model while being amenable to analytical treatment [21]. The integrate-and-fire neuron is a particular case of the SRM.

The considered neuron receives inputs through multiple synapses indexed by 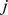, and the incoming spikes received through each of these synapses during the considered trial are indexed by 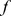 according to their temporal order. We consider that the arrival of the 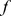-th presynaptic spike on the synapse 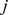 of a neuron at the moment 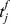 leads to a postsynaptic potential (PSP) whose value as a function of the time 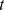 is the product of synaptic efficacy 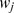 and a normalized kernel 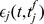, i.e. 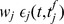 (Methods). We consider here that synaptic changes are applied on a time scale that is much slower than the time scale of the variation of the PSPs and than the length of the considered trial, or that, in simulations, synaptic changes are applied at the end of one or more trials grouped in batches of information processing within which synaptic efficacies are held constant. Thus, the synaptic efficacies 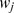 can be considered effectively constant during a trial, but can change across trials. We denote as 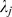 the total normalized PSP resulting from the contribution of past presynaptic spikes coming through the synapse 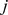,
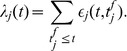
(1)The membrane potential 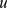 of the neuron is determined by the integration of the PSPs generated by all presynaptic spikes, and also by a term 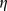 that models the refractoriness caused by the last spike fired by the studied neuron:
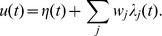
(2)When the membrane potential reaches the firing threshold 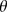, a spike is fired and the membrane potential is reset to the reset potential 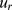.

**Figure 1:**
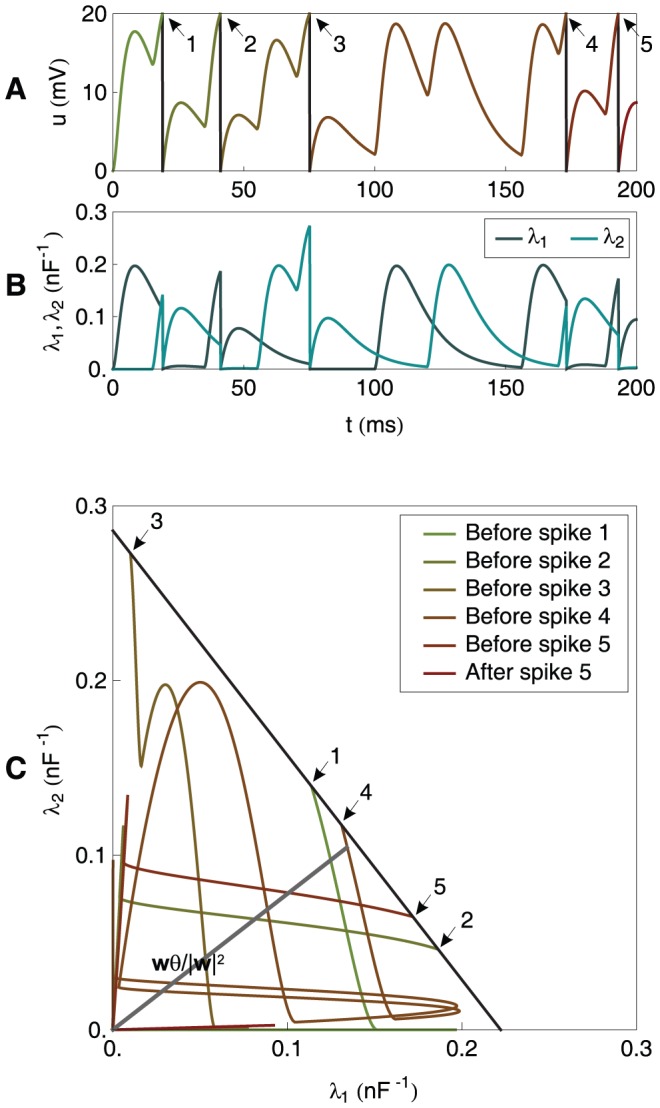
A graphical illustration of the chronotron problem for a neuron with 2 synapses. (A) The dynamics of the membrane potential 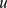. The numbered arrows indicate the timings when the membrane potential reaches the firing threshold and spikes are fired. (B) The dynamics of the two components of 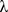. (C) The trajectory of 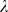. Spikes are generated when the trajectory reaches the spike-generating hyperplane, which is here a line. The chronotron problem is solved by adjusting the location of the spike-generating hyperplane, through changes in 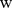, such that the timings of the fired spikes are the target ones. The numbered arrows indicate the generation of spikes at the times when the spike-generating line is reached. The neuron has 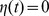.

**Figure 2:**
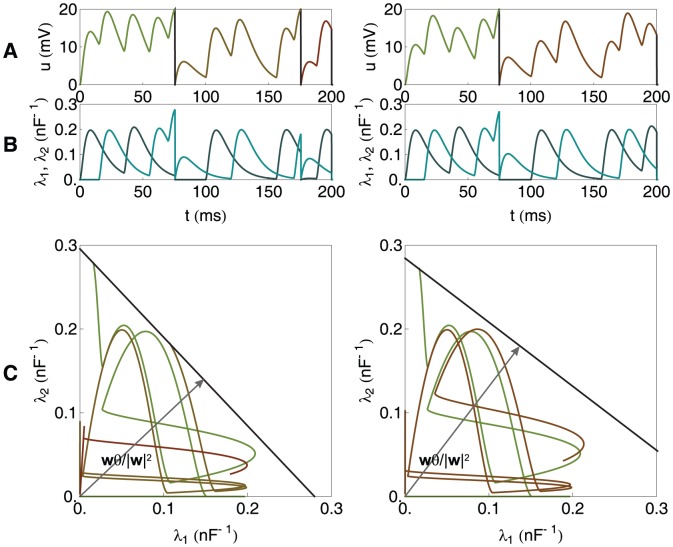
A graphical illustration of the chronotron problem for a neuron with 2 synapses (continued). As in Fig. 1, but for other values of 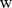, resulted through the application of E-learning, starting from the situation in Fig. 1, and having as a target the generation of one spike at 75 ms. Left: during learning. Right: after learning converged.

**Figure 3:**
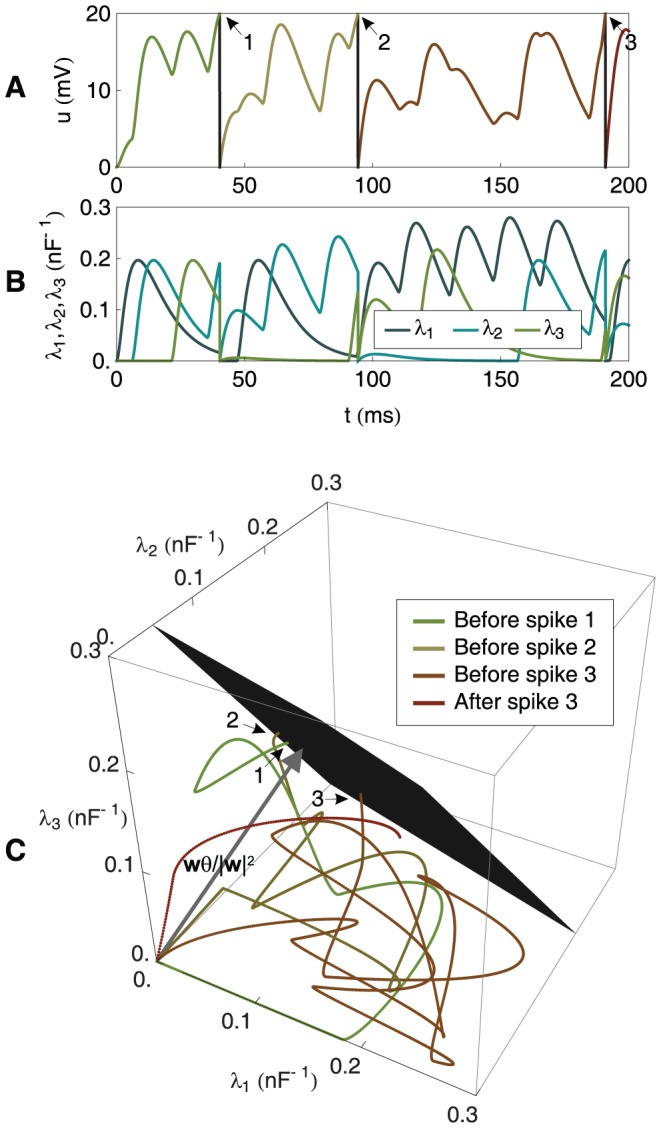
A graphical illustration of the chronotron problem for a neuron with 3 synapses. (A) The dynamics of the membrane potential 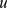. The numbered arrows indicate the timings when the membrane potential reaches the firing threshold and spikes are fired. (B) The dynamics of the three components of 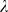. (C) The trajectory of 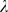. Spikes are generated when the trajectory reaches the spike-generating hyperplane, which is here the black plane. The numbered arrows indicate the generation of spikes at the timings when the spike-generating hyperplane is reached. The neuron has 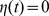.

The chronotron problem can be illustrated graphically by considering a space having the same number of dimensions 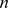 as the number of afferent synapses of the neuron. In this space, the 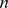 synaptic efficacies 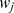 define a vector 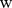 and the normalized PSPs 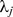 define a vector 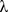. The vector 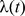 moves around this space, in time, according to the dynamics of the PSPs, while 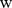 changes on much larger timescales than 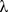. The neuron fires when 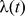 touches a hyperplane that is perpendicular on 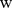 and at a distance 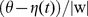 of the origin (Methods). After firing, the PSPs are reset to 0 and thus the trajectories of 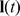 always start from the origin. This is illustrated in Figs. 1 and 2 for a neuron with 2 synapses and in Fig. 3 for a neuron with 3 synapses. The chronotron problem can be understood as the problem of setting the spike-generating hyperplane, by changing 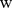, such that it intersects the trajectory of 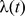 at exactly those timings when we want spikes to be fired. This problem is very similar to the problem that needs to be solved in reservoir computing [22]–[24], where the state of a high-dimensional dynamical system, such as our vector 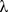, is processed by a (usually) linear discriminator such that the switch between output states (the crossing of the hyperplane defined by the linear discriminator) happens at desired moments of time.

Similar optimization problems can usually be solved by defining an error function and then changing the parameters to be optimized, through methods like gradient descent, which minimize this error function. The differences between the actual spike train fired by the neuron for a particular input and, respectively, the target spike train can be measured using spike train metrics such as the Victor & Purpura (VP) distance [25]. The VP distance is defined as the minimum cost for transforming one spike train into the other by creating, removing or moving spikes [25]. However, one cannot derive an efficient learning rule using directly this distance, because the terms corresponding to spikes that should be created or removed are constant and do not reflect how creating or removing these spikes depends on the plastic parameters. In order to solve this issue, we used a new error function, which is a modification of the VP distance.

### E-learning

The VP distance is the sum of the costs assigned to either insertion of spikes, removal of spikes or shifting the timing of spikes. The cost of adding or deleting a single spike is set to 1, while the cost of shifting a spike by an amount 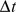 is 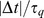, where 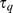 is a positive time constant that is a parameter of the metric. Instead of constant cost terms for the independent spikes that have to be created or removed, our error function changes the VP distance by including terms that depend on the value of the membrane potential of the neuron at the timings of these spikes. This allows these terms to be differentiated piecewisely with respect to the plastic parameters (Methods).

For a given input, the trained neuron fires at the moments 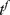, where 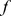 represents the index of the spike in the spike train. The ordered set of the spikes in the spike train fired by the neuron is 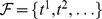. The target spike train that the neuron should fire for that input is 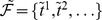. In a transformation of minimal cost, according to the VP metric, of the actual spike train 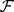 into the target one 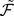, the operations involved are the following: removal of spikes (that are not previously moved); insertion of spikes (at their target timings, so that they are not moved after insertion); and shifting of spikes toward their target timings. We denote as 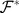 the subset of 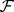 that represents the spikes that should be eliminated; and as 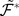 the subset of 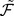 that represents the timings of target spikes at which new spikes should be inserted into 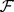. We call the spikes in 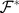 and 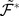 independent. The spikes in the actual spike train that are not eliminated, 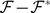, are in a one-to-one correspondence with the spikes in the target spike train for which a correspondent is not inserted, 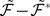, and they should be moved towards their targets. We say that the spikes in correspondent pairs from 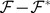 and 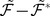 are linked or paired to their correspondent (match). The existing algorithm that computes the VP distance between two given spike trains [25] can be extended in order to also compute the sets 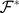, 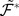 and their complements (Methods).

**Figure 4:**
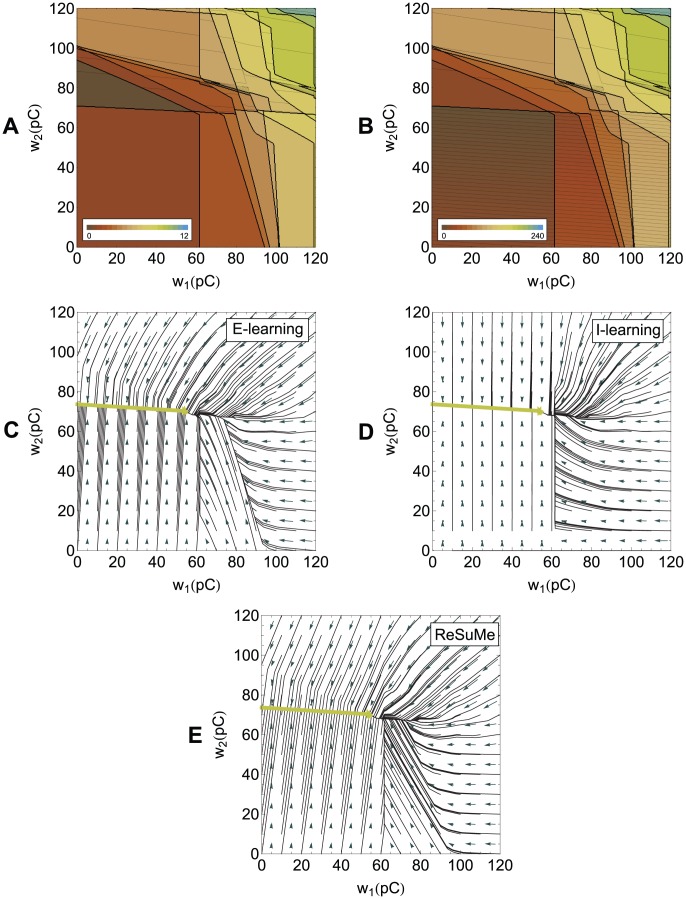
The error landscape for a neuron with two synapses and the descent on this landscape during learning. The neuron receives several input spikes on each synapse, the same as in Figs. 1 and 2, and has to fire one spike at a predefined target timing, the same as in Fig. 2. (A), (B) A contour plot of the VP and E distances between the actual spike train and the target spike train as a function of the values of the synaptic efficacies. The thick lines correspond to discontinuities of the distances. (A) VP distance. (B) E distance. (C), (D), (E) The dynamics of the synaptic efficacies according to the learning rules. The black lines represent actual trajectories of the synaptic efficacies. The vectors represent synaptic changes. The green line corresponds to the values of the synaptic efficacies for which the output corresponds to the target spike train. (C) E-learning. (D) I-learning. (E) ReSuMe.

E-learning aims to minimize the following error function:
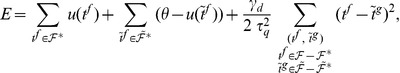
(3)where 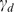 is a positive parameter. The first sum runs over the independent actual spikes, the second sum runs over the independent target spikes, and the last sum runs over all unique pairs of matching spikes. Because the creation and deletion of spikes and changes in their classification in either 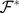 or 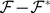 lead to discontinuous changes of 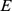 (Fig. 4 B), gradient descent can only be ensured piecewisely. The synaptic changes that aim to minimize the error function through piecewise gradient descent are 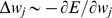. By performing the derivation and after some approximations (Methods), we get the E-learning rule:
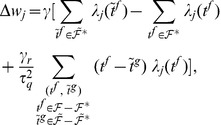
(4)where 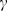 is the learning rate, a positive parameter, and 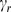 another positive parameter.

**Figure 5:**
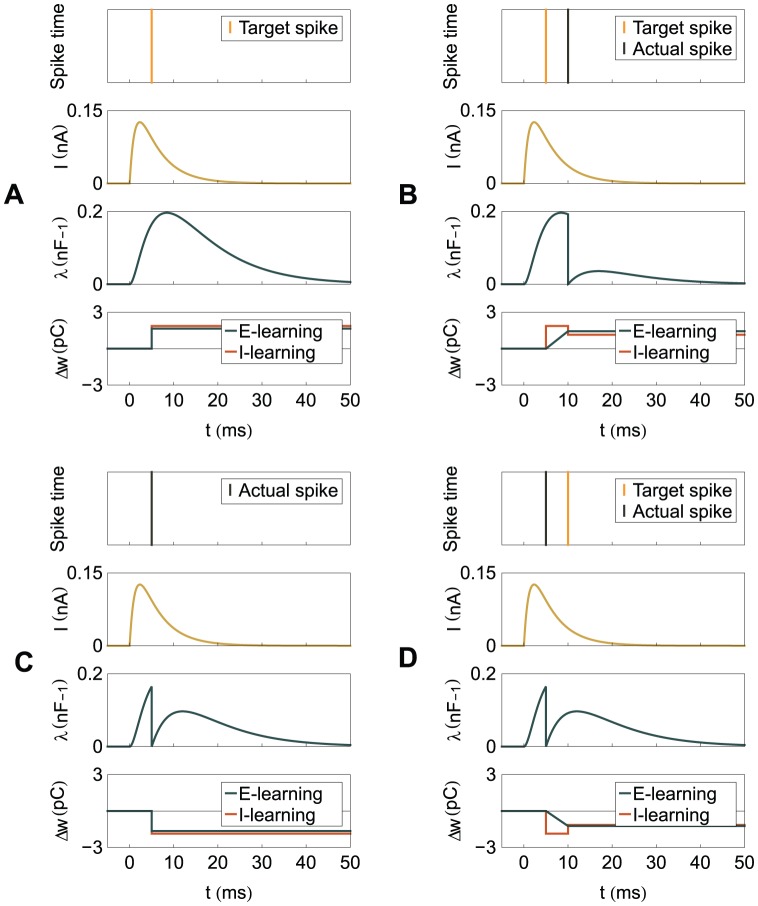
A graphical illustration of the plastic changes implied by the learning rules. The graphs show the spike timings and, for one synapse, the dynamics of the synaptic current 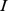, the normalized PSP 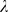 and the synaptic changes 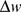 implied by the two learning rules. It is considered that one input spike arrives at this synapse at 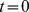. The synaptic changes are shown to be localized temporally along the events that cause them; the actual application of the synaptic changes can be delayed with respect to these events. (A) One independent target spike and no actual spike. (B) A pair of matching target and actual spikes, the actual one following the target one. (C) One independent actual spike and no target spike. (D) A pair of matching target and actual spikes, the target one following the actual one.

E-learning works by modifying each synaptic efficacy 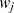 by terms that depend on the normalized PSP 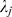. For all target spikes that the neuron should fire, for which a spike should be created, each synaptic efficacy should be increased with a term proportional to 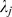 at the moments of these target spikes. For all output spikes that should be eliminated, each synaptic efficacy needs to be decreased with a term proportional to the value of 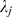 at the moments of these spikes. For all actual spikes that are close to their target positions and should be moved towards them, each synaptic efficacy needs to change with a term proportional to the value of 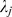 at the moments of the actual spikes, multiplied by the temporal difference between actual and target spikes. Fig. 5 illustrates the learning rule.

The E-learning rule is appropriate for both excitatory and inhibitory synapses. If we consider that the excitatory synapses have a positive synaptic efficacy 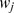 and the inhibitory synapses have a negative one, the learning rule in the form presented above can be applied to both cases. Without an extra bounding of the synaptic efficacies, E-learning will transform an excitatory synapse into an inhibitory one or viceversa, as needed for performing the task.

E-learning aims to minimize the error function by performing piecewise gradient descent. The inherent discontinuities introduced in the error function by creation or removal of spikes or by creation or breaking of matching pairs of actual and target spikes may possibly lead to both increases and decreases of the error function. However, the terms that reflect in the error function spikes that should be created or removed ensure that the membrane potential is increased or, respectively decreased at the corresponding timings, such that the number of spikes becomes the desired one and the actual spikes are close to the target ones. Because the learning rule uses approximations, it is possible that gradient descent is not ensured, not even piecewisely. Thus, the optimality of E-learning cannot be guaranteed analytically. However, as the simulations have shown, E-learning is more efficient for chronotron training than the other existing learning rules, having a much higher memory capacity.

It is possible to devise a continuous error function that is then properly derivable, yielding a proper gradient descent that can be guaranteed analytically. However, the continuous error function would be much more complex than the current one, yielding a complex learning rule. This would encumber an intuitive understanding of the learning rule, as it is possible with E-learning. A learning rule based on a continuous error function will be presented elsewhere.

### I-learning

The second learning rule that we developed is heuristic and is inspired by both the E-learning rule and the existing ReSuMe learning rule [19], [20] (Methods). The I-learning rule is defined by
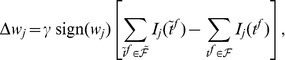
(5)where 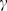 is the learning rate, a positive parameter, and 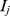 is the synaptic current on the synapse 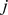. In our simulations, synaptic currents were modeled as a difference between two exponentials (Methods).

As in ReSuMe, actual and target output spikes lead to synaptic changes of equal amplitude but of opposite signs, such that when the actual spike train corresponds to the target one the terms cancel out and synapses become stable. In ReSuMe, synaptic changes depend exponentially on the relative timings of pairs of pre- and postsynaptic spikes, as in some models of spike-timing-dependent plasticity. In contrast, here we consider that synaptic changes depend on the value of the synaptic current at the timings of spikes. This learning rule is thus biologically-plausible, since it depends on quantities that are locally available to the synapse. Target spikes determine synaptic potentiation, while actual spikes lead to synaptic depression. We call this synaptic current-dependent rule I-learning (Fig. 5).

Because 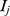 is proportional to 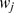, synaptic changes under I-learning converge to zero when 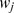 approaches zero, for small 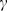. Thus, the I-learning rule does not allow an excitatory synapse to become inhibitory or viceversa. This corresponds to how neurons in the brain release neurotransmitters that lead, for a particular presynaptic neuron, to either excitation or inhibition of postsynaptic neurons having potentials not far from the resting potentials (Dale's principle) [26].

In E-learning, synaptic changes caused by activity within a trial can be computed only at the end of the trial, because one needs the actual spikes fired during the entire trial under study in order to compute which spikes are independent and which are linked, although one can imagine approximate algorithms for matching the spike trains, which would also work online. In I-learning, synaptic changes can also be applied online, which is more biologically relevant.

### Performance of the learning rules

**Figure 6:**
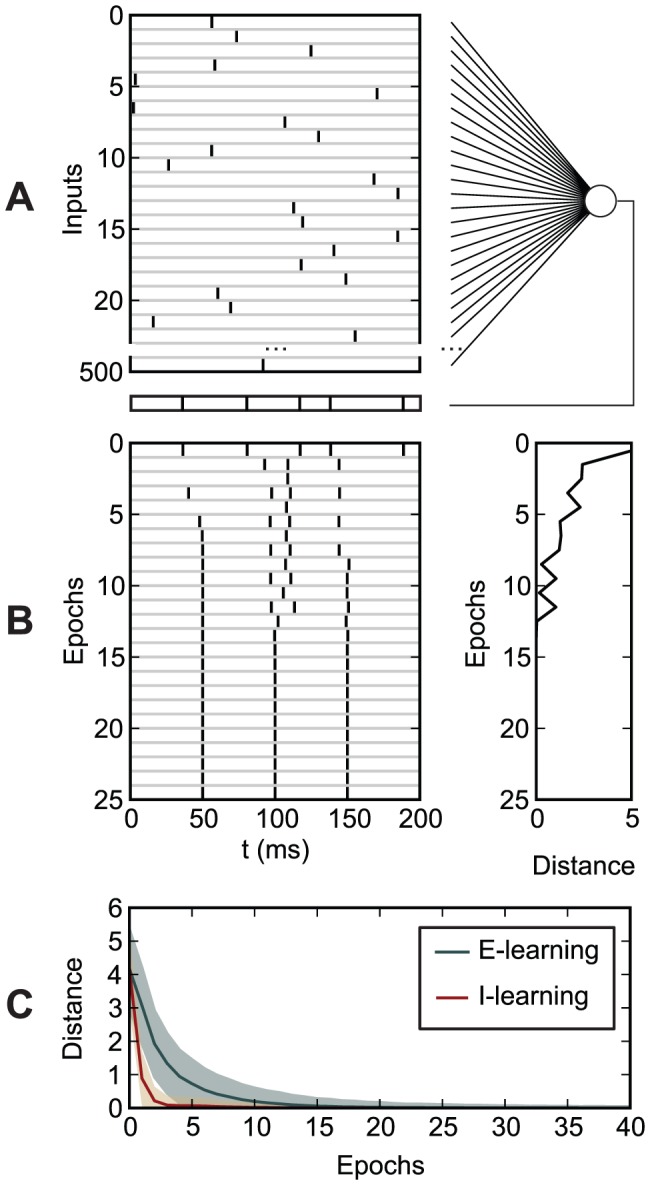
Learning of a mapping between one input pattern and one output spike train. The trained neuron receives inputs from 500 neurons. The spike trains received from these neurons form the input pattern. Each input spike train consists of one spike within the 200 ms of a trial, generated at a random timing having an uniform distribution within the trial. The target output spike train consists of spikes at 50, 100 and 150 ms. (A) Part of the input pattern and the output spike train of the trained neuron, corresponding to this input, before learning. Only some of the 500 input spike trains are illustrated. (B) The synaptic efficacies change according to E-learning, such that the trained neuron's output reproduces the target spike train. Left: The output spike train during learning. Right: The VP distance between the actual and the target output spike train, during learning. The target output is reproduced after less than 15 epochs (presentations of the input pattern). (C) The VP distance between the actual and the target output spike train during learning, for E-learning and I-learning: averages and standard deviations over 10,000 realizations of the same experiment. Each realization uses different, random input spike trains and initial values of the synaptic efficacies.

We have studied these rules in computer simulations involving integrate-and-fire neurons. Both learning rules allow a neuron to perform temporally-accurate input-output mappings. Fig. 6 illustrates learning of a mapping between one input pattern (the spike trains coming through all input synapses) and one output spike train consisting of three spikes. The learning rules perform a descent in the landscape defined by the VP or E distance (Fig. 4).

We studied next setups where the chronotron had to memorize multiple input-output associations. Both the input and the output encoded information in the precise spike timings: both input and output spike trains consisted of one spike per trial and the timing of this spike represented the information (time-to-first-spike coding or latency coding). The length of spike patterns (and of one simulation trial) was 200 ms. The latency of a spike with respect to the beginning of a trial can correspond to the phase of a spike with respect to a background oscillation, modeling a phase-of-firing encoding of information, and multiple trials can correspond to multiple periods of the oscillation. This could model experimentally-observed situations where phase locking of spikes relative to a theta rhythm is associated to encoding and memorizing of information [6], [9], [10], [13], [14].

**Figure 7:**
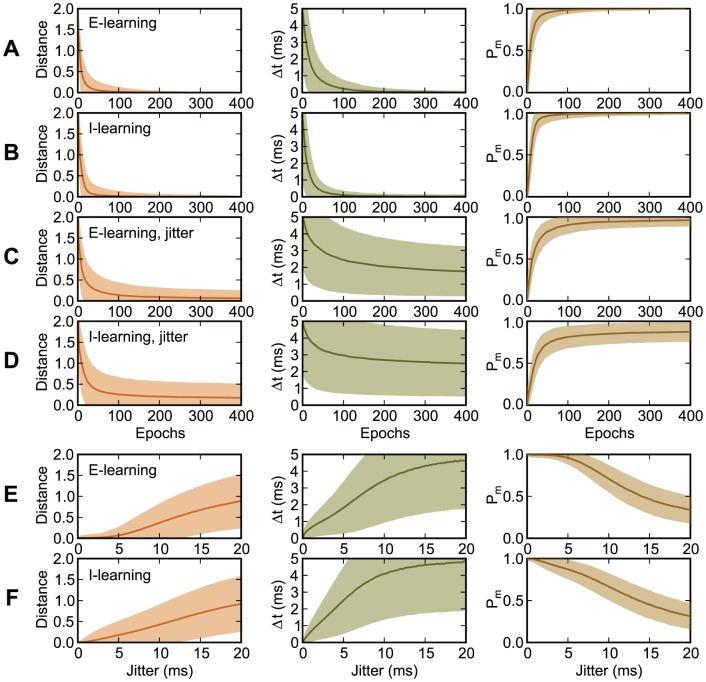
Learning of a mapping between 10 input patterns, with and without jitter, and one output spike train. Left: The VP distance between the actual and the target output spike train. Center: The timing difference 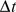 between matching spikes and the target spikes. Right: The probability 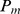 that the fired spikes matched the target ones. The graphs represent averages and standard deviations over input patterns and over 10,000 realizations. (A)–(D): Evolution during learning, as a function of the learning epoch. (A), (B): No jitter. (C), (D): A gaussian jitter with an amplitude of 5 ms is added to each presentation of the input patterns. (E), (F): Values after 400 learning epochs, as a function of the amplitude of the input jitter. (A), (C), (E): E-learning. (B), (D), (F): I-learning. The inputs and the trial length are as in Fig. 6. The target output spike train consists of one spike at 100 ms.

Fig. 7 illustrates learning of a mapping between 10 different input patterns and one output spike train consisting of one spike at the middle of the trial interval. The neuron learns to perform this mapping, for all 10 input patterns, using the same set of synaptic efficacies. For example, for E-learning, in 99.9% of 10,000 realizations, the neuron was able to fire the correct number of spikes (one spike) and the spike had an average timing difference of less than 0.03 ms with respect to the timing of the target spike, after about 8 minutes of learning (simulated time; 241 learning epochs). In 95% of realizations, the average timing error was less than 1 ms after 1.6 minutes of learning (48 learning epochs). Learning worked even when the inputs were jittered, i.e. at each trial, input spikes were displaced around the reference timing according to a gaussian distribution. For example, in the same conditions as before but with an input jittered with a 5 ms amplitude, in more than 95% of the realizations, the neuron fired one spike with an average timing error of less than 2 ms, after about 8 minutes of learning (225 epochs). A 5 ms gaussian jitter amplitude corresponds to a 3.99 ms average timing displacement of the input spikes (Methods), so, in this case, the mapping also led to noise reduction, by doubling the precision of spike timing.

**Figure 8:**
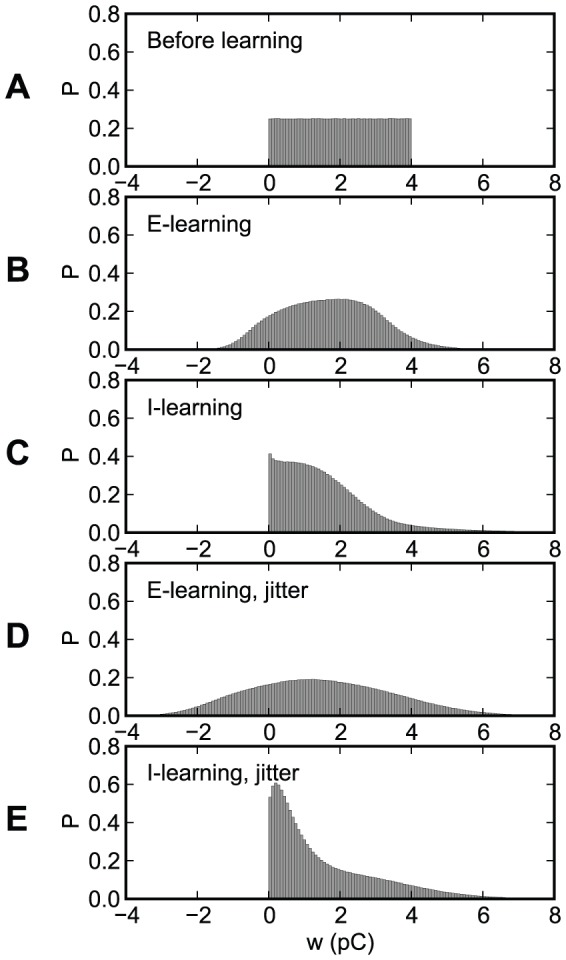
The distribution of the synaptic efficacies, before and after learning, for the experiments presented in **Fig. 7**. (A) Before learning. (B)–(E) After 400 learning epochs. (B), (D) E-learning. (C), (E) I-learning. (B), (C) No jitter. (D), (E) A gaussian jitter with an amplitude of 5 ms is applied to the inputs.

Fig. 8 presents the distribution of the synaptic efficacies, before and after learning, for the experiments presented in Fig. 7. This distribution has been computed over the 10,000 realizations of the experiments. With I-learning, all synapses stay excitatory, like they were generated initially, although a significant fraction of them become close to zero, after learning. E-learning allows synapses to change sign. When the input is subject to jitter, the synaptic distributions after learning become broader than in the case of no jitter.

### Memory capacity of the chronotron

The chronotron is able to perform generic classification tasks, where 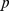 input patterns must be classified into 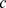 categories through hetero-association. For all the different input patterns in one category, the chronotron must fire the same output spike train, using the same set of synaptic efficacies. In our simulations, equal number of patterns were randomly assigned to each category.

**Figure 9:**
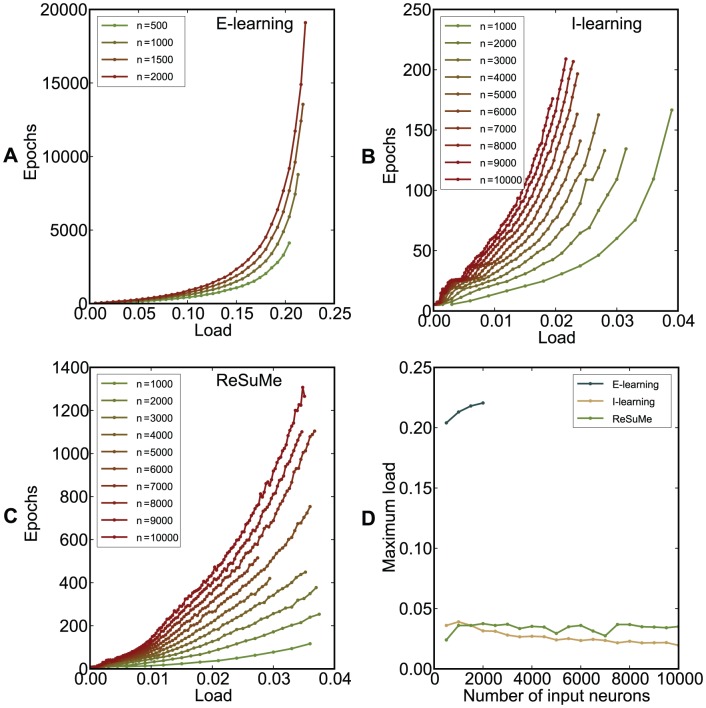
The performance of the chronotron learning rules for a classification problem. The input patterns are classified into 3 classes. (A)–(C) The average minimum number of epochs required for correct learning is displayed as a function of the load 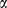, for various values of the number of input synapses 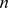. Note the scale differences. (A) E-learning. (B) I-learning. (C) ReSuMe. (D) The maximum load for which correct learning can be achieved (the capacity 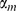), as a function of 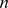. E-learning has a much better performance than I-learning or ReSuMe. For E-learning, simulations for higher 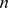 were not performed because of the high computational cost, due to the high capacity resulted through this learning rule. Averages were computed over 500 realizations with different, random initial conditions.

The ability of neurons to memorize mappings corresponding to classification tasks increases with the number of input synapses 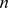. The ratio 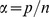 (the number of input patterns memorized per input synapse) represents the load imposed by the task on the neuron. A characteristic of the neuron's ability to learn is the maximum load for which the mappings are performed correctly [16], which we call the capacity 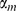 of the neuron. We considered that the chronotron had a correct output when target spikes were reproduced with a 1 ms precision, which corresponds to the lower end of the 0.15–5 ms range of the precision of spikes observed in several areas of the brain [27]–[34]. In our setup, in both input and target output spike trains there was one spike per trial and information was encoded in the spike latencies. Except where specified, the input spike trains consisted, for each of the 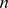 synapses, of one spike generated at a random timing, distributed uniformly, and the target spike train for each category 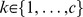 consisted of one spike at 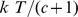 (Methods). Fig. 9 illustrates the performance of the chronotron in simulations where inputs were classified into 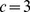 categories. For the particular studied setup, both I-learning and ReSuMe led to a capacity between 0.02 and 0.04, while E-learning led to a capacity up to 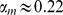 patterns per synapse.

**Figure 10:**
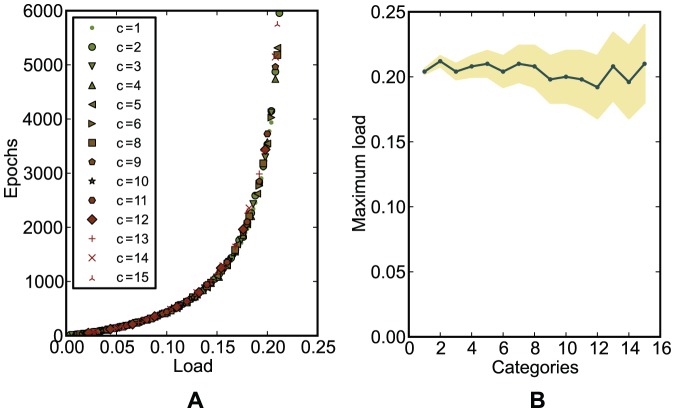
The dependence on the number of categories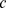 of the performance of E-learning for a classification problem. (A) The average minimum number of epochs required for correct learning, as a function of the load 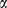, for various numbers of categories 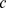. Regardless of 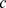, the points fall on the same curve. (B) The maximum load for which correct learning is achieved (the capacity 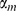), as a function of the number of categories 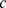. The shaded area represents the uncertainty due to the fact that the load can vary only discretely, in steps of 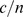, for a particular 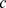. The capacity is approximately constant for all 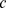.

The load and the capacity have been used to characterize neurons with binary outputs, which memorize one bit of information for every pattern. The chronotron can classify inputs in more than one category, and for 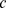 categories it memorizes 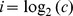 bits of information for every input pattern. Therefore, a better measure for the chronotron's learning ability is the information load 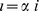 and the corresponding information capacity 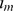, equal to the maximum information load. The number of categories into which a chronotron with latency coding of information classifies its inputs is limited only by the temporal precision of the output spike. For example, if this temporal precision is 1 ms, with the particular setup presented here, the chronotron can encode up to about 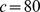 categories (Methods). When information is encoded in the spike latencies, the simulations showed that the chronotron's capacity does not depend on the number of categories 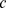 (Fig. 10). The maximum information capacity of the chronotron, for E-learning and the particular setup that we used, can be then computed as 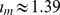 bits per synapse (Methods). Extrapolating, this means that a chronotron with about 10,000 input synapses would be able to fire a spike at the correct timing, with a 1 ms precision, among up to 80 possible ones, for about 2,200 different, random input patterns, and thus to memorize about 13.9 kilobits of information. The information capacity of the perceptron is 2 bits per synapse and the one of the tempotron is around 3 bits per synapse [16]. However, if more than two input categories have to be discriminated, the chronotron has the advantage of being able to carry computations that need multiple perceptrons or tempotrons to be performed, being thus more efficient. Unlike the tempotron, the chronotron uses the same coding of information for both inputs and outputs and is therefore able to interact with other chronotrons.

The capacities computed here for the chronotron are lower bounds, since it might be possible to develop learning rules which are more efficient than E-learning and to devise setups with more efficient encoding of information.

### Dependence on the setup parameters

**Figure 11:**
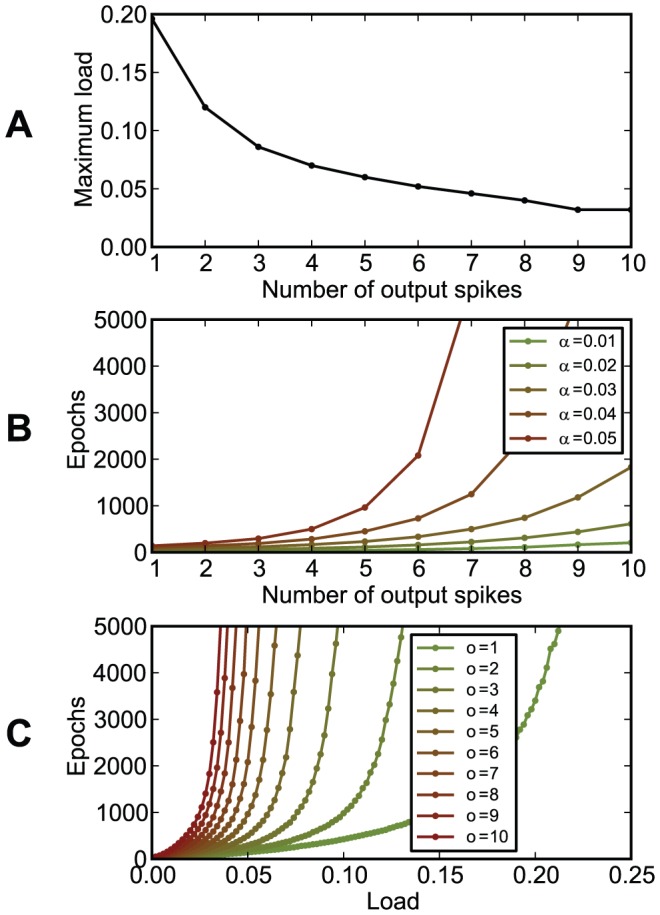
The dependence of chronotron performance on the number of output spikes per trial. The neuron had to learn to have the same output for all inputs, using E-learning. The output consisted of 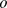 output spikes, placed at 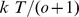, for 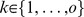. (A) The maximum load (the capacity 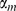) as a function of the number of output spikes 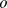. (B) The number of learning epochs required for correct learning as a function of the number of output spikes 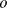, for various loads 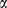. (C) The number of learning epochs required for correct learning as a function of load, for various numbers of output spikes 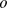. Best performance was achieved for a single output spike per trial.

**Figure 12:**
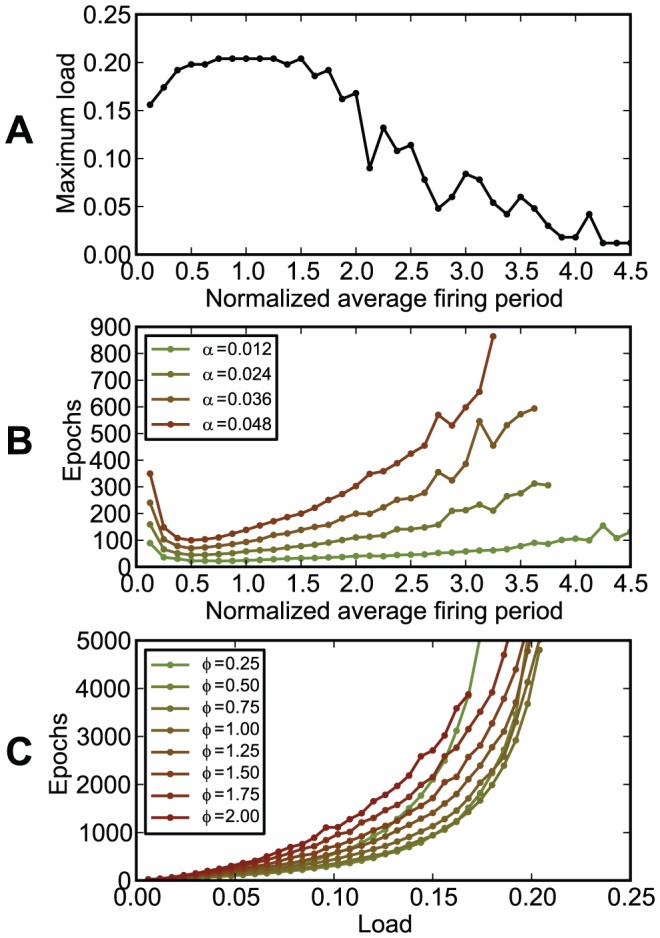
The dependence of chronotron performance on the firing rate of the inputs. The inputs were generated using a Gamma process having a normalized average period (the average period over the trial length) 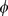 (Methods). (A) The maximum load (the capacity 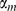) as a function of the normalized average period 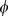. (B) The number of learning epochs required for correct learning as a function of the normalized average period 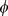, for various loads 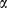. (C) The number of learning epochs required for correct learning as a function of load 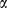, for various values of the normalized average period 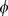. Best capacity was achieved for values of 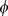 around 1, i.e. a single input spike per trial, for each synapse, on average, while fastest learning was achieved for 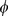 around 0.5.

**Figure 13:**
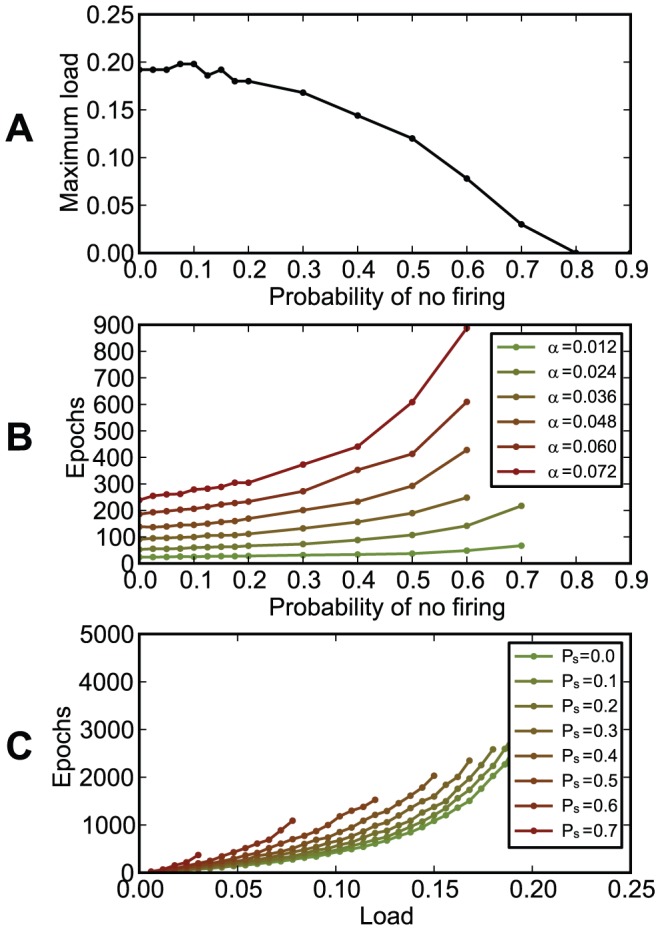
The dependence of chronotron performance on the probability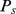 that input synapses receive no spikes. At the beginning of the experiment, each input spike train was set up as either one spike generated at a random timing or, with a probability 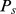, of no spikes. Input patterns did not change during learning. (A) The maximum load (the capacity 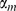) as a function of the no firing probability 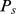. (B) The number of learning epochs required for correct learning as a function of the no firing probability 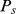, for various loads 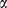. (C) The number of learning epochs required for correct learning as a function of load 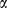, for various values of the no firing probability 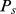. Best capacity was achieved for values of 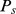 less or equal to 0.1, while fastest learning was achieved when there was no input with no spikes. For large 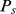 there are not enough input spikes to drive the neuron and, as expected, performance drops.

In our setups, information was represented in the precise timings of spikes relative to the beginning of trials of constant duration. If trials correspond to periods of a background oscillation, the timing of spikes corresponds to the phase relative to this oscillation. Simulations performed in this framework have shown that chronotrons have the best efficacy when both input and output spike trains consist of one spike per trial (period). Setups where inputs or outputs consisted of more than one spike, or where some of the inputs fired no spikes, had suboptimal performance in terms of learning speed and memory capacity (Figs. 11, 12, and 13). However, learning was still possible under all of these conditions, unless the input pattern included too few spikes (less than about 100, for our setup).

**Figure 14:**
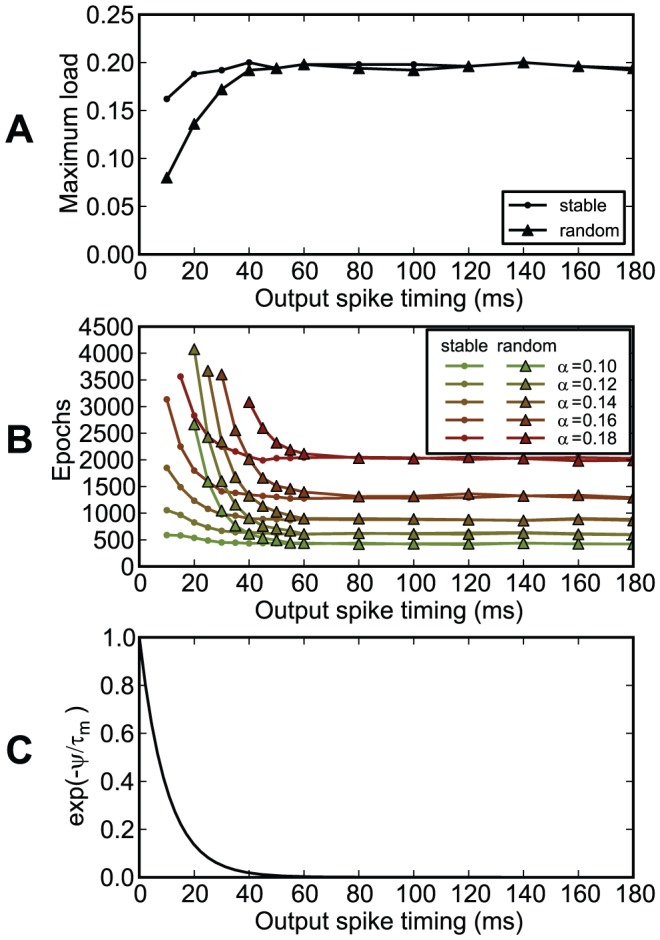
The dependence of chronotron performance on the timing of the output spike and on the initial state of the membrane potential. The neuron had to learn to have the same output for all inputs. The output was one spike at a given timing 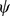. At the beginning of each trial, the membrane potential 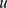 was either set to 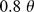, as in the other experiments (stable initial state), or was generated randomly, with a uniform distribution, between 0 and 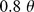 (random initial state). (A) The maximum load (the capacity 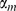) as a function of the timing of the output spike 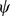. (B) The number of learning epochs required for correct learning as a function of the timing of the output spike 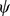, for various loads 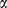. (C) 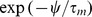, as a reference for comparing the effect on learning of the initial conditions, as a function of the timing of the output spike 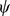. For this setup, the capacity and the learning time for reaching the correct output, for stable initial state, does not depend on 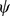 if it is larger than about 40 ms. Because of the exponential decay of the membrane potential of the chronotron with a time constant 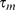, the effect of the random initial state of the membrane potential on the chronotron's performance, as a function of the output spike timing 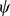, becomes insignificant at about 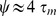, similarly to 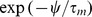, as 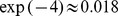.

Chronotron's efficacy was not affected by the initial state of their membrane potential at the beginning of trials if target spike times were set at a delay relative to the beginning of the trial of more than about 4 times the time constant of the membrane potential's exponential decay (Fig. 14).

**Figure 15:**
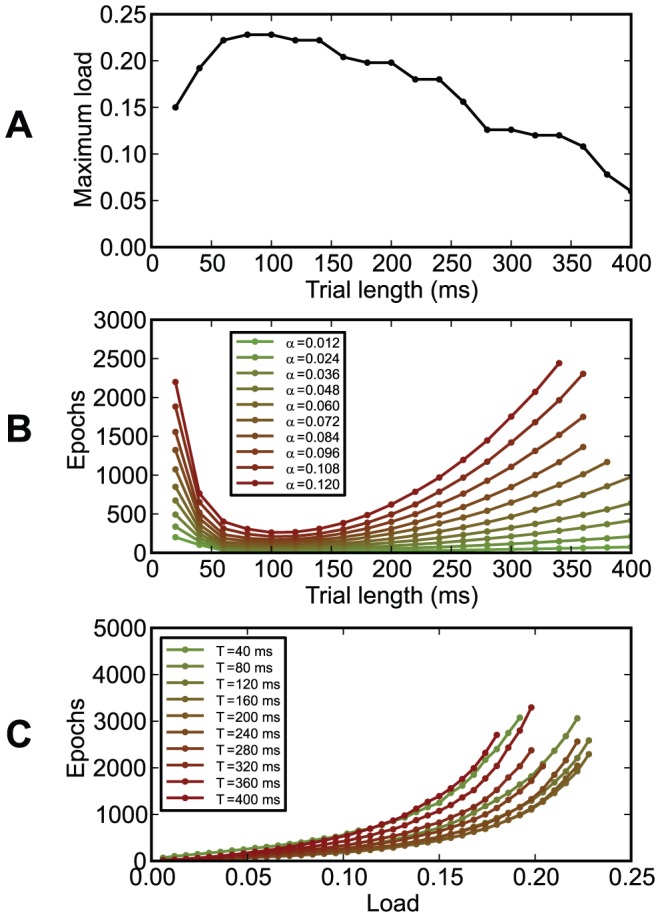
The dependence of chronotron performance on trial length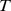. (A) The maximum load (the capacity 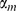) as a function of the trial length 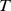. (B) The number of learning epochs required for correct learning as a function of the trial length 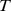, for various loads 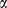. (C) The number of learning epochs required for correct learning as a function of load 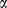, for various values of the trial length 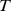. Best performance was achieved for 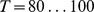 ms (the relevant parameter is 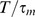, 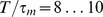).

In our setup, the chronotron had an optimal memory capacity if the trial length (the oscillation period) was about 8–10 times larger than the membrane time constant (Fig. 15). Since typical neurons in the brain have membrane time constants between 8 and 100 ms [35]–[40], this would correspond to oscillation periods between 64 and 1000 ms (frequencies between 1 and about 16 Hz), an interval that covers the theta rhythm.

**Figure 16:**
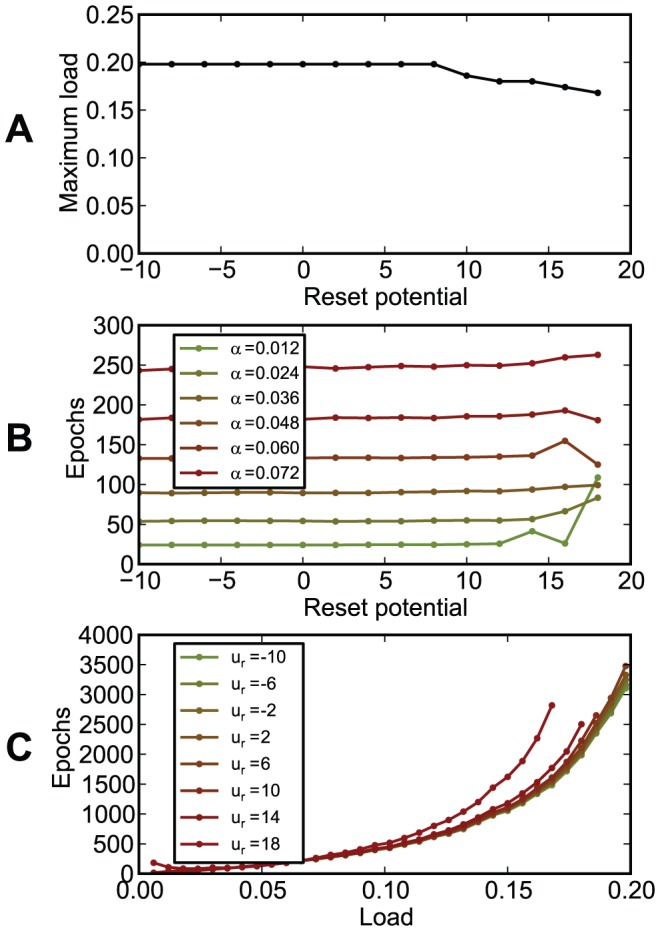
The dependence of chronotron performance on the reset potential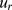. (A) The maximum load (the capacity 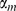) as a function of the reset potential 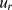. (B) The number of learning epochs required for correct learning as a function of the reset potential 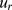, for various loads 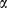. (C) The number of learning epochs required for correct learning as a function of load 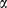, for various values of the reset potential 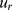. The performance does not depend on the reset potential if it is lower than half of the firing threshold, 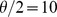 mV.

The chronotron's performance did not depend on the reset potential if it was lower than half of the firing threshold 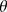 and declined slowly for higher reset potentials, which are, however, artificially high (Fig. 16).

**Figure 17:**
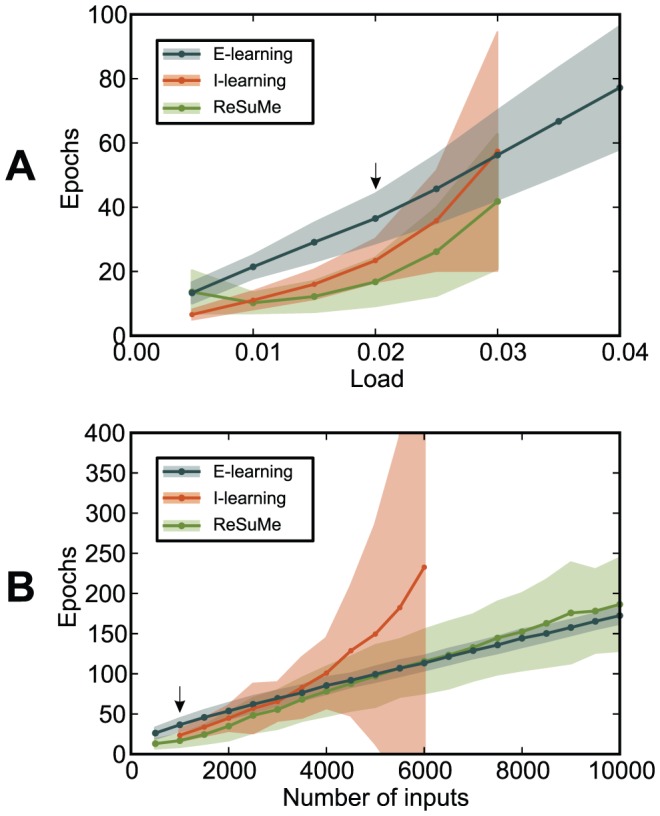
The performance of learning rules when their parameters were optimized for fast learning for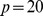, 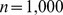 (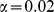). (A) The number of learning epochs required for correct learning as a function of the load 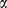, for 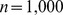. Correct learning was not achieved for I-learning and ReSuMe for 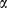 larger than 0.03. (B) The number of learning epochs required for correct learning as a function of the number of input synapses 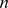. Correct learning was not achieved for I-learning for 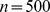 nor 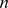 larger than 6,000. Averages and standard deviations over 500 realizations. The arrows indicate the conditions for which the parameters were optimized.

In Fig. 17, parameters were optimized to lead to the minimum average number of learning epochs needed for correct learning for a setup with a relatively low load, 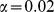. For the setup that was optimized and for the optimal parameters, ReSuMe had the fastest learning (16.75
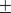
7.43 epochs), followed by I-learning (23.39
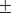
6.87 epochs) and E-learning (36.48
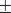
7.61 epochs). However, the advantages of the first two learning rules over E-learning disappeared for setups with higher loads or higher number of input synapses than the optimized setup, when the other parameters were kept the same.

**Figure 18:**
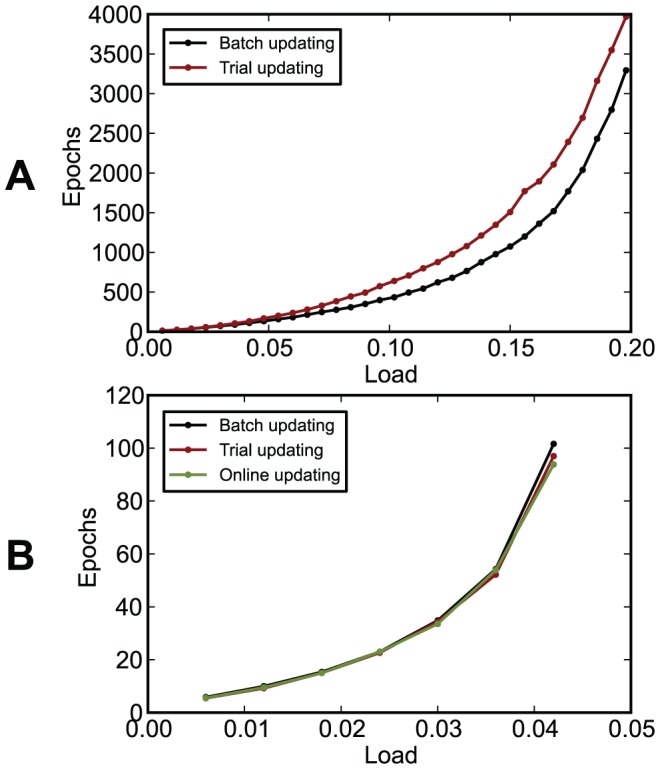
The dependence of chronotron performance on when synapses are updated during simulations. The number of learning epochs required for correct learning as a function of the load 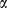, for various methods of applying the synaptic changes according to the learning rules: batch updating (synapses are changed at the end of each batch of 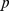 trials, each one corresponding to one of the input patterns); trial updating (synapses are changed at the end of each trial); online updating (synapses are changed after each target or actual postsynaptic spike — for I-learning only). (A) E-learning. (B) I-learning.

In our simulations, the synaptic changes defined by the learning rules were accumulated and were applied to the synapses at the end of each batch consisting of 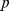 trials (presentations of the 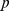 input patterns) [41], [42]. Simulations of E-learning where synaptic changes were applied at the end of each trial required a slightly higher number of epochs for correct learning, but led to the same memory capacity (Fig. 18 A). Simulations of I-learning where synaptic changes were applied either at the end of each trial or online, triggered by postsynaptic spikes (as in Fig. 5) did not lead to results significantly different than simulations with batch updating of the synapses (Fig. 18 B).

### Comparison to other results

The first supervised learning method for spiking neurons was SpikeProp [43], [44], a method inspired by the backpropagation algorithm used for training classical neural networks. SpikeProp works by minimizing the difference between the timing of an output spike and the desired timing. The first versions of the learning method required a feedforward network and that each neuron in the network fires only once during a trial. Later versions [45]–[51] extended the method for including a momentum term; adapting the synaptic delays, time constants and neurons' thresholds during learning; for networks where the input (but not the output) neurons fire more than once per trial; for recurrent networks; and for improving learning speed under certain assumptions. However, the method is designed for adjusting just the timing of a single (first) spike per output neuron and assumes that the synapses are such that each output neuron fires at least one spike for the given inputs. The method is not suitable for adjusting the number of output spikes nor for training a neuron to fire given output spike patterns that extend beyond the first spike.

Carnell and Richardson [52] devised a method for modifying the synaptic weights such as the weighted sum of the presynaptic spike trains (in an algebraic representation) converge to a desired one. If the neuron model is such that the firing of the postsynaptic neuron is close to this weighted sum, then the method allows the supervised learning of a target output spike train. The method is quite original and general, but ignores the details of the dynamics of the postsynaptic potential and of the neuronal membrane.

Pfister et al. [53] have derived supervised learning rules for probabilistic neurons. The learning method is based on gradient ascent in the space of synaptic efficacies, which maximizes the likelihood of having a trained neuron firing at the desired moments. Because of the probabilistic framework, the learning rules do not involve the actual timing of the output spikes, but the probability of having a particular output spike train given a particular input spike train. Calculating such a probability while taking into account the reset of the membrane potential after the spikes of the output neuron is computationally challenging and not biologically plausible.

Legenstein and colleagues [54], [55] have studied a supervised, biologically-inspired learning method for spiking neurons that works by clamping neurons to the desired output and applying spike timing–dependent plasticity (STDP) to the afferent synapses of the trained neurons. Under certain conditions, after learning, the neurons yield the desired output even after the teaching signal is removed. The effectiveness of this learning method has been proved analytically only for Poisson input spike trains, and there are worst case scenarios where the method fails, but simulations have shown that the method is effective in more general conditions. The method works only when synapses have hard bounds, by driving synaptic efficacies toward these bounds. Thus, the output patterns that this method can learn are restricted to those that can be generated by synapses that have either minimum (zero) or maximum efficacy. A similar rule can be used for supervised learning of patterns by networks [56], but not by single neurons.

The tempotron [16] implements supervised learning for a particular task where an output neuron either fires one spike or does not fire during a predetermined time interval, when presented with an input spike pattern that encodes information in the precise spike timings. The approach assumes that after the neuron emits a spike in response to a input pattern all other incoming spikes have no effect at all on the neuron (are shunted), which is artificial. The timing of the output spike cannot be controlled with this method, and thus the output of a tempotron cannot be used as an information-carrying input for another tempotron. The tempotron has a binary response and therefore its output cannot distinguish between more than two input categories. Although it is claimed that it is biologically plausible, the tempotron learning rule requires information that is nonlocal in time, needing to monitor the maximum of the output, and information that is not available to the neuron, such as the maximum of the membrane potential that would have been reached if the neuron would have not fired. We have shown that the tempotron is equivalent to a particularization of the ReSuMe learning rule [17]. A learning rule by Urbanczik and Senn [18] improves the original tempotron learning rule but is still focused on the artificial tempotron setup.

Barak and Tsodyks [57] have developed learning rules that increase the variance of the input current evoked by a set of learned patterns relative to that obtained from random background patterns. The trained neuron then has a larger firing rate when presented with one of the learned patterns, as compared to when presented with a typical background pattern. The learning rules are quite complex, with low biological plausibility. They allow a neuron to recognize input patterns of precisely timed spikes, but the timing of the output spikes is not controlled by these rules. The memory capacity computed for these learning rules, for just the recognition of patterns, is an order of magnitude smaller than the maximum memory capacity we obtained for mapping memorized patterns to specific outputs (Methods). Other complex setups for recognizing spike patterns were also developed [58]–[61].

A few other supervised learning methods for spiking neurons or neural networks also exist but work only for some specific cases, such as neurons receiving oscillatory inhibition [62], population-temporal coding [63], theta neurons [64], [65], neurons with very large membrane decay time constants and constant interspike intervals for the inputs [66], networks with time to first spike coding for classification through plasticity of synaptic delays [67], neurons having two presynaptic and one postsynaptic spikes per learning cycle [68], specific configurations, composed of several modules, of the trained network [69].

ReSuMe [19], [20], [70]–[74] is a general supervised learning method for spiking neurons that allows learning of arbitrary output spike trains. It is the only existing learning rule that is comparable to the ones introduced here. This learning rule has been conjectured by analogy to the Widrow-Hoff rule for analog neurons. Simulations have shown that not all the terms of the conjectured learning rule are needed for learning [74]. To date, it has been shown analytically that ReSuMe will converge to an optimal solution only for the case of one input spike and one target output spike [70]. We have shown here (Fig. 9) that E-learning leads to a much higher memory capacity than ReSuMe. The higher performance of E-learning can be attributed to the analytical derivation of the E-learning rule, although the derivation included approximations that preclude analytical guarantees on the optimality of E-learning.

I-learning is quite similar to ReSuMe. As in ReSuMe, in I-learning actual and target postsynaptic spikes lead to synaptic changes of opposite signs, such that when the actual spike train corresponds to the target one the terms cancel out and synapses become stable, and thus the basic mechanism is identical. In contrast to the typical form of ReSuMe, where synaptic changes depend exponentially on pairs of pre- and postsynaptic spikes, as in some models of spike-timing-dependent plasticity, in I-learning synaptic changes depend on the value of the synaptic current. In the case that synaptic currents are exponentials, I-learning would be identical to a form of ReSuMe where the non-Hebbian terms are set to zero. Variants of ReSuMe where the exponentials have been replaced by other types of functions, including differences between two exponentials (double-exponentials) like in our model of I-learning, have been previously studied [74] but these double-exponentials have not been previously associated to the synaptic currents. ReSuMe is typically presented as using exponential functions and non-zero non-Hebbian terms [20]; the lack of these in I-learning makes it distinct from ReSuMe. Because the rising part of the double-exponentials is deleterious to learning [74] and because I-learning does not allow synapses to change sign, unlike ReSuMe, I-learning has, in most cases, a lower performance than ReSuMe (Figs. 9, 17). However, unlike in the experiment with double-exponentials in [74], where, additionally to the terms that we used in I-learning some anti-Hebbian terms have been used and a lack of convergence has been observed, in our experiments I-learning converged well to the target output (Fig. 6, 7). Real synaptic currents do involve a non-zero rising time and thus using double-exponentials in modeling currents is biologically relevant.

For a review of supervised learning methods for spiking neural networks, see [75].

## Discussion

We have shown that, through appropriate learning methods, spiking neurons are able to process and memorize information that is encoded in the precise timing of spikes. We presented two new spike-timing-based learning rules, E-learning and I-learning, which allow neurons to fire specific spike trains in response to specific input patterns of spike timings, by modifying accordingly their synaptic efficacies. E-learning leads to high memory capacity, while I-learning is more biologically plausible.

There obviously are input-output mappings that are mechanistically impossible to be performed by a spiking neuron. For example, when there is no input, the neuron obviously cannot fire. A sufficient number of input spikes that arrive uncorrelated on each of its synapses leads to a wide range of outputs that the neuron is able to map to these inputs, by adjusting the synaptic efficacies. But if the neuron has to perform several different input-output mappings with the same set of synaptic efficacies, the various mappings constrain each other through the synaptic efficacies. These constraints lead to the mechanistical impossibility that the neuron performs new input-output mappings beyond the current ones, and thus to a finite memory capacity of the neuron. We computed lower bounds of the memory capacity of a spiking neuron with temporal coding of information and studied how this depends on various parameters of the setup.

The chronotron can model situations where information is coded in the time of the first spike relative to the onset of salient stimuli [2], or situations where information is coded in the phase of spiking relative to a background oscillation. Some of the results presented here underline the role of oscillations for temporal information processing. First, oscillations segment time into frames (periods), offering a reference for temporal encoding of information in spike latencies (phases) [76]. Second, in the parts of the cycles where neurons are globally inhibited or global excitation is low, oscillations ensure that neurons are reset such that they are able to process independently the inputs corresponding to different frames (periods). If this reset is such that it allows the chronotron to get into the resting state or another baseline state (the chronotron is inhibited or does not receive significant input for a duration of about 4–6 times the membrane time constant or more), then the absolute latencies, relative to the oscillation period, of the input spikes do not matter for the chronotron, but just the relative timings of spikes in the input pattern. The output spikes of the chronotron would then encode information in their relative timings with respect to the input spikes. The relevance of oscillations to temporal coding is consistent to the results of Havenith et al. [8] where the information carried by neurons in the visual cortex through their relative firing times was found to increase considerably with the oscillation strength. It has also been shown that the hippocampal theta rhythm is necessary for learning by rats of the Morris water maze [77] and that it enhances learning in eyeblink classical conditioning in rabbits [78], [79]. Oscillations also enhance the temporal precision of action potentials [80]. Although the parameters of most of our simulations correspond to a theta rhythm, the simulations' results remain the same when all temporal parameters are rescaled, and thus the results are also relevant for neurons that are subject to a gamma rhythm or other oscillations.

In the brain, when the spike phase encodes information relative to a background oscillation, the neurons fire no more than one spike per cycle in some, but not all, experiments [4], [6], [8]–[10], [14]. Our results showed that firing one spike per cycle is optimal for processing and memorization of phase-of-firing temporally encoded information by spiking neurons. In many cases, neurons in the brain skip oscillation cycles, which implies that the neurons that skip cycles do not participate in all input patterns received by postsynaptic neurons. This means that postsynaptic chronotrons will have an effective number of inputs lower than the real one, which would reduce the memory capacity as compared to the case when all input neurons fire one spike per cycle. However, this does not preclude the possibility that chronotrons process and memorize information in oscillatory networks where neurons skip cycles or where neurons fire more than one spike per cycle.

I-learning implies that synaptic changes are proportional to the corresponding synaptic currents, which are quantities that are locally available to the synapse. Postsynaptic spikes lead to synaptic depression similar to anti-Hebbian spike timing-dependent plasticity (STDP) [81]–[89], while the timings of target postsynaptic spikes trigger potentiation. The depression and potentiation should balance each other when actual spikes occur at the target timings. The target timings could be indicated by spikes coming from other, teacher neurons, through special teaching synapses [90]. The firing of these teacher neurons should lead to heterosynaptic associative changes [91]–[93] according to the I-learning rule and should not have a significant impact on the trained neuron's potential [90]. The potentiation generated through such a mechanism should be balanced by anti-Hebbian STDP when the trained neuron reproduces the firing of the teacher neuron. In this case, the trained neuron's firing should then become increasingly correlated to the one of the teacher neuron, eventually mimicking its firing with a lag corresponding to the delay of the arrival of the teaching spikes. If the trained neuron learns from several teacher neurons, it should learn to fire when either one of the teacher neurons fires, acting thus as a kind of multiplexer. If the trained neuron does not need to reproduce the entire activity of teaching neurons, but just the one during salient events, teaching could be modulated by a neuromodulator. Neuromodulation of supervised learning could be similar to the control of induction of associative plasticity in Purkinje cells through targeted modulation of instructive climbing fiber synapses [94] or the neuromodulation of STDP [95]–[102]. Just as STDP [103]–[105] or its neuromodulation [106], [107] were predicted theoretically in advance of experimental verification, future experiments may find plasticity mechanisms similar to I-learning in the brain. For example, such mechanisms might be responsible for neural synchronization that modulates neural interactions [108], such as the synchronization of thalamic neurons needed for driving the cortex through weak synapses [109]; for encoding of information through synchronization [110]; or for the fine temporal tuning of excitation relative to inhibition that contributes to stimulus selectivity in rat somatosensory cortex [111].

Besides the particular supervised learning rules introduced here, other learning mechanisms, such as reinforcement learning, or developmental mechanisms selected through evolution, could lead to a chronotron-like processing of temporally-coded information.

Many computational applications of the presented learning rules are possible. For example, the supervised learning rules presented here could be used to train readout neurons of liquid state machines, for which perceptrons or spiking neurons with rate coding of outputs were previously used [23]. Using spiking neurons with temporal coding as readouts for liquid state machines makes their information representation compatible to the one of spiking neurons in the liquid, thus allowing the outputs of the readouts to be fed back into the liquid. Such feedback significantly improves the computing power of liquid state machines [112], allowing the development of better models of information processing in the brain. Another possible application is the decoding of neural signals. Less efficient learning rules than the ones presented here have been already applied successfully, and with better results than alternative methods, to train simulated spiking neural networks to extract arm movement direction and hand orientation intent from the timing of spike trains recorded from monkeys [113]. These are just a few examples of the potential uses of the learning rules presented here. These rules open the way to a plethora of future experiments that will explore how information encoded in the precise timing of spikes can be processed and memorized. This should lead to a better understanding of the information-processing features of neurons in the brain.

## Methods

### The neural model

Our analysis uses the Spike Response Model (SRM) of spiking neurons, which reproduces with high accuracy the dynamics of the complex Hodgkin-Huxley neural model while being amenable to analytical treatment [21], [114]. For this model, the dynamics of the membrane potential 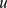 of a neuron as a function of the time 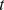 is given by
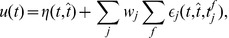
(6)where 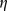 is a kernel that represents the refractoriness caused by the last spike of the neuron; 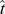 is the last time the neuron fired before 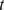; the first sum runs over all synapses 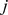 afferent to the considered neuron; 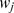 is the synaptic efficacy of the synapse 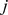; the second sum runs over the set of the timings when spikes coming through synapse 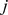 reach the postsynaptic neuron, 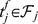; 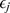 is a normalized kernel that determines the postsynaptic potential (PSP) caused by a presynaptic spike. We consider here that synaptic changes are applied on a time scale that is much slower than the time scale of the variation of the PSPs and than the length of the considered trial, or that, in simulations, synaptic changes are accumulated and applied at the end of one or more trials grouped in batches of information processing within which synaptic efficacies are held constant. Thus, the synaptic efficacies 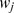 can be considered effectively constant during a trial, but can change across trials. We have chosen the reference of the membrane potential such that the resting potential of the neuron is 0. The 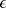 kernel is causal, i.e. 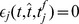 for 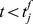, and also decays to 0 for 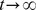. We denote as 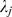 the total normalized PSP resulting from the contribution of past presynaptic spikes coming through the synapse 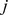,
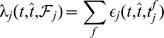
(7)and therefore
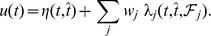
(8)When the membrane potential reaches the firing threshold 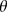, the neuron fires a spike and the membrane potential is reset.

### Graphical illustration of the chronotron problem

We consider the problem of training the plastic parameters of a spiking neuron, such that the spike train of the trained neuron is, for a given input, as close as possible to some given target spike train. Although we focus here on training the synaptic efficacies, the plastic parameters may also be the synaptic delays, the firing threshold, the membrane time constant, etc.

We consider the vector 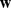 having as components the synaptic efficacies 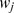 and the vector 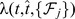 having as components the normalized PSPs 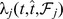. Each of these vectors has a dimension equal to the number of synapses, 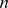. The equation that defines the dynamics of the Spike Response Model, Eq. 8, can be then rewritten in vectorial form as
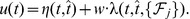
(9)


The dynamics of the normalized PSPs define a trajectory of 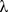 in the corresponding 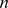-dimensional space. After each postsynaptic spike, the normalized PSPs are reset to 0 and thus this trajectory always starts from the origin of the space. The neuron fires a spike when 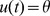, or
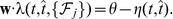
(10)


The magnitude of the projection of the 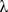 vector on the 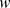 vector is 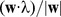. Thus, the neuron fires a spike when the magnitude of the projection of 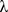 on 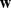 reaches 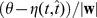, i.e. 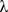 reaches a spike-generating hyperplane which is perpendicular on the vector 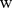 and at a distance 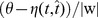 of the origin. This is illustrated in Figs. 1 and 2 for a neuron with 2 synapses and in Fig. 3 for a neuron with 3 synapses. These artificially low numbers of synapses were chosen because it is difficult to visualize spaces with dimensions higher than 3.

The chronotron problem can then be understood as setting the vector 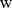 such that the spike-generating hyperplane that it defines is such that 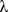 reaches it at the moments of the target spikes.

### Analytical derivation of the E-learning rule

For a given input, the trained neuron fires at the moments 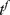, where 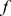 represents the index of the spike in the spike train. The ordered set of the spikes in the spike train fired by the neuron is 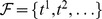. The target spike train that the neuron should fire for that input is 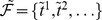.

The key to solving the chronotron problem is finding appropriate error functions that can be afterwards minimized through methods like gradient descent in the space of the plastic parameters. In order to find such an error function, we start from the Victor & Purpura (VP) family of metrics based on spike times that defines distances between pairs of spike trains [25], [115]. The distance between two spike trains is defined as the minimum cost required to transform one into the other. This is the sum of the costs assigned to either insertion of spikes, removal of spikes or shifting the timing of spikes. The cost of adding or deleting a single spike is set to 1, while the cost of shifting a spike by an amount 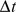 is 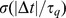, where 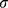 is a positive, increasing function with 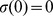, and 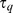 is a positive time constant that is a parameter of the metric. The commonly used form of this function is simply 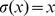 [25], [115].

Because the transformation is of minimal cost, the operations that define it are severely constrained. The same spike cannot be both moved and deleted, nor inserted and moved, nor inserted and deleted. A spike can be moved in only one direction, and the trajectories of moved spikes should not intersect [25]. Thus, in a transformation of minimal cost of the actual spike train 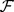 into the target one 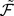, the operations involved are the following: removal of spikes (that are not previously moved); insertion of spikes (at their target timings, so that they are not moved after insertion); and shifting of spikes toward their target timings. The order of these operations is irrelevant.

We denote as 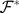 the subset of 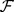 that represents the spikes that should be eliminated; and as 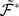 the subset of 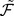 that represents the timings of target spikes at which new spikes should be inserted into 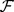. The spikes in the actual spike train that are not eliminated, 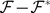, are in a one-to-one correspondence with the spikes in the target spike train for which a correspondent is not inserted, 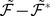, and they should be moved towards their targets. We say that the spikes in 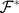 and 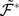 are independent, while the spikes in 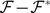 and 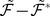 are linked or paired to their correspondent (match). The VP distance is then
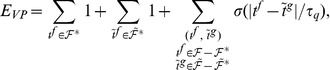
(11)where the first sum equals the number of elements in 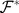, the second sum equals the number of elements in 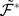, and the last sum runs over all unique pairs of matching spikes.

The existing algorithm that computes the VP distance between two given spike trains [25], [115] can be extended in order to also compute the sets 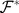, 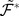 and their complements. We present this extended algorithm in a separate section, below.

Thus, we can determine which of the actual spikes fired by the trained neuron should be removed, which target spikes do not have a correspondent and thus new spikes should be created to match them, and which spikes should be moved and toward which of the targets, in order to transform the actual spike train into the target one with a minimal cost. The plastic parameters should then change in order to perform this transformation.

For an existing spike at 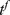 that should be moved towards 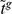, the error that should be minimized is 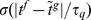. This can be differentiated piecewisely with respect to the plastic parameters, so that the changes of the parameters that lead to a decrease of the error can be computed. However, the spikes in 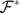 and 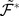 that are independent contribute to the distance a constant term of 1 each, and this is not differentiable with respect to the plastic parameters. In order to be able to minimize the contribution of these terms to the distance between the spike trains, we must focus more closely on the mechanisms of spike creation and removal.

The neuron fires a spike when its membrane potential 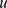 reaches the firing threshold 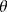; after a spike is emitted, the membrane potential is reset to 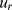. If a new spike should be created at a target timing 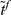, this is because the membrane potential is not high enough at that moment. In order to minimize the spike train distance by creating a new spike, we should thus minimize the error 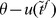. This reflects the amount with which the membrane potential should increase at 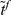 in order to reach the threshold and let the neuron fire at the target timing. Analogously, if an actual spike at 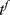 should be removed we should decrease the membrane potential at that timing and minimize 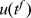. Note that we minimize the membrane potential at the *current* moments of the spikes to be removed. The membrane potential at a *generic* moment of these spikes equals the firing threshold, thus being a constant that cannot be minimized. The effect of this minimization will be, in most cases, a change of the timing of these spikes, until their elimination.

These error terms that depend on the values of the membrane potential at the timings of the spikes are piecewisely differentiable with respect to the plastic parameters. We will replace, in the error function to be minimized by changes in the plastic parameters, the constant terms corresponding to independent actual and target spikes with these new error terms. Because the new error terms are not commensurable with the original spike train distance, we scale the original terms by a constant, positive parameter 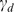. The final error function that we seek to minimize is thus
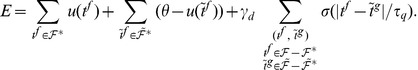
(12)The first sum is over the independent actual spikes, the second sum is over the independent target spikes, and the last sum is over unique pairs of linked spikes, consisting of one target spike and one actual spike that should be moved towards the target one.

We aim to minimize this error function by piecewise gradient descent in the space of the plastic parameters of the trained neuron. We will consider here training the efficacies 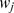 of the synapses afferent to the neuron, where the index 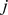 indicates the synapse. The synaptic changes that aim to minimize the error function are thus
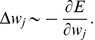
(13)


We have
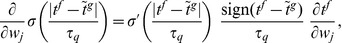
(14)where 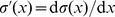. Because of the presence of the absolute value function in the argument of 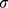, the derivative presented in the equation above is discontinuous when the actual spike is at its target timing, 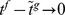, unless we have 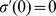. We would like to fulfill this condition in order to avoid, during learning, oscillations of the emitted spikes around the target positions. The typically used linear function 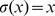 does not fulfill this condition. Because of this, here we will use
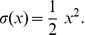
(15)Like for the commonly used form 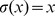, for our choice of 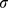 the switch from considering two spikes (one from each of the two spike trains) as independent to considering them as linked is when the difference of their timings, in absolute value, is 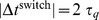. This is because a pair of independent spikes contributes to the distance with a term of 1 each, for a total of 2 (one actual spike should be removed and a matching spike for the target one should be created); and 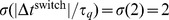.

We have 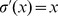 and
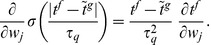
(16)The derivative of the firing time of the neuron with respect to a synaptic efficacy can be computed by taking into consideration that the firing time depends on the synaptic efficacies through its dependence on the dynamics of the membrane potential of the neuron. However, the membrane potential at a generic firing time is always constant, equal to the firing threshold, and thus we have [44], [116]:
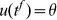
(17)

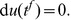
(18)By expanding the last equation, we get
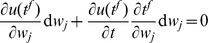
(19)and, finally,
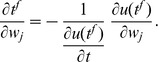
(20)


By introducing Eqs. 12, 16, and 20 into Eq. 13, we get:
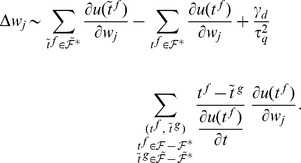
(21)


In order to be able to compute the derivatives of the membrane potential with respect to the synaptic efficacies, we have to choose a specific neural model. As discussed above, here we use the Spike Response Model, Eq. 8. We can then compute
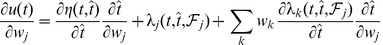
(22)

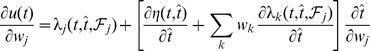
(23)

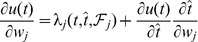
(24)


In order to simplify the learning rule, its presentation and its computational implementation, we neglected the last term in the last equation and we used for the simulations the approximation
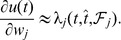
(25)The neglected term is not necessarily numerically negligible, but however the simulations have shown that the learning rule performs well under this approximation.

Another approximation that we used was to replace the factor 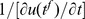 in Eq. 21 with a constant. This is needed because this factor diverges numerically when a spike is fired while the membrane potential barely reaches the threshold and 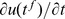 is close to 0. This divergence reflects a discontinuity of the studied system [48]: in this situation, an infinitesimally small change of a synaptic efficacy can lead to a finite change of the error function, if this results in the removal of the considered spike. Our error function deals with spike creation or removal trough the two terms that ensure that the membrane potential is increased or, respectively, decreased at the desired timings, such that the number of spikes becomes the desired one and the actual spikes are close to the target ones. It is thus safe to enforce a hard bound for the divergent factor or, as we did here, to replace it with a constant. This constant is positive, because a spike is generated only when the membrane potential increases. We fold this constant and 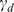 into a new positive constant, 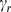.

The resulting learning rule, that we call E-learning, is thus
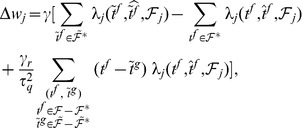
(26)where 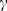 is the learning rate, a positive parameter.

The E-learning rule can be described more intuitively as follows. For each of the target spikes, if these target spikes are independent (do not have a corresponding actual spike close to them), each synapse 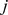 is potentiated proportionally to the normalized PSP 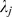 at the moments of these target spikes. For each of the independent actual spikes (that do not have a corresponding target spike close to them), each synapse is decreased proportionally to the normalized PSP at the moments of these actual spikes. For each pair of matching spikes, each synapse changes proportionally to the difference between the timing of the actual spike and the timing of the target spike in the pair, and also proportionally to the normalized PSP at the moment of the actual spike. The first two terms of the learning rule will create or remove spikes in order to match them to the target ones. The last term of the learning rule will move the actual spikes that match the target ones toward their targets. When the timing of the spikes coincide to their targets, the changes of the synaptic efficacies suggested by the learning rule become zero and thus learning stops.

The E-learning rule can also be understood intuitively by considering snapshots of the trajectory of 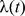 at the timings of target and actual spikes of the trained neuron. The equation defining the E-learning rule, Eq. 26, can be written in vectorial form as
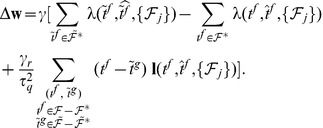
(27)At the timings 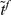 of independent target spikes, the spike-generating hyperplane must be brought closer to the 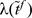 vector and therefore the 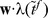 product must be increased. This can be done best, for a given perturbation of 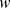, by increasing just the component of 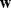 that is parallel to the 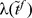, which would lead 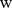 to turn towards 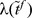. This leads to setting 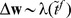, hence the first term of Eq. 27. At the timings 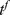 of independent actual spikes, 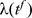 reaches the spike-generating hyperplane which must be then moved away from 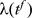 and thus it leads to 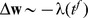, hence the second term of Eq. 27.

When an actual spike at 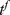 is followed closely by a matching target spike at 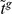, bringing the spike-generating hyperplane closer to 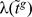 is deleterious since it does not take into account that 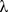 has just been reset to 0 because of the recent actual spike. In this case, what should be done is just delaying the actual spike. This could be done by moving the spike-generating hyperplane away from 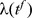, proportionally to 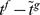. When a target spike at 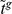 is followed closely by a matching actual spike at 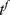, bringing the spike-generating hyperplane closer to 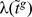, in the same way as in the case of an independent target spike described above, would bring the timing of the actual spike closer to the target one, but in an imprecise fashion. We would like that the convergence of the actual spike towards the target one to be smooth. The third term of Eq. 27, 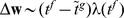, takes care of the last two situations.

### The I-learning rule

The form of the synaptic changes indicated by the previously described E-learning rule depends on whether spikes are independent or not, being different in the two cases. While, as the simulations have shown, this learning rule is very efficient, the biological plausibility of this switch of the form of the synaptic changes is debatable. For this reason, we sought a more biologically plausible supervised learning rule.

We consider the limit 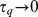, when all spikes are independent: 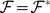 and 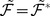. In this case, the E-learning rule will keep just its first two terms, which depend on the normalized postsynaptic potentials. The switch of the form of the synaptic changes as a function of the pairing status of the spikes is then removed. However, due to the spike generation mechanism, the postsynaptic potentials suffer a discontinuity after each actual spike, being reset to zero. If there is no distinct treatment of pairs of close actual and target spikes, this leads to a discontinuity of the synaptic changes when an actual spike oscillates around a target one. Learning does not converge to a stable firing of the actual spikes at the target timings. Moreover, it is not clear whether the normalized postsynaptic potential (i.e., the postsynaptic potential with the synaptic efficacy factored out) is a quantity locally available to the synapse.

For these reasons, we heuristically defined a new learning rule. As before, we wanted the synaptic changes to depend on a quantity that reflects the contribution of each synapse to the membrane potential, a quantity that would be correlated to 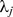, which is used by the analytically-derived E-learning rule. As in E-learning with 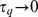, the synaptic changes for excitatory synapses should be determined by synaptic increases proportional to the value of the considered quantity at the timing of the target spikes and by synaptic decreases proportional to the value of that quantity at the timing of the actual spikes. In this case, when the actual spikes coincide with the target ones, the terms cancel out, resulting in the convergence of the learning rule. Another condition was that the sum of the terms corresponding to a pair of close actual and target spikes converges continuously to zero when the actual spikes moves towards the target one. We also wanted that the quantity used by the learning rule to be locally available to the synapse, thus ensuring its biological plausibility. We thus used the synaptic current, 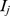, as this quantity. The resulting learning rule, that we call I-learning, is thus:
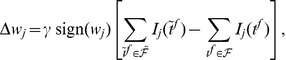
(28)with 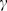 being the learning rate, a positive constant. Although we did not make it explicit in the notation, the synaptic currents 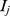 on each synapse obviously depend on the parts of the presynaptic spike trains 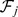 coming through that synapse previous to the moment at which 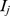 is evaluated. The 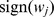 in the learning rule (i.e., 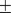
1 as a function of whether the synapse is excitatory or inhibitory) reflects that the sign of the synaptic changes depends on the sign of the synaptic efficacy. For excitatory synapses, both 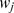 and 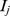 are positive, while for inhibitory synapses both are negative.

### ReSuMe

We have also performed simulations using the ReSuMe learning rule [19], [20], [73], [74], in order to compare it to the new learning rules introduced here. We used the following form of ReSuMe [20]:
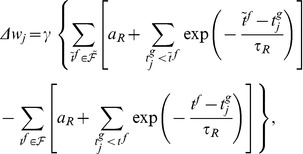
(29)where 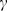 is the learning rate, 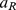 is a non-Hebbian term, and 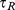 is a time constant, all being positive parameters of the learning rule.

### Details of the neural model

**Figure 19:**
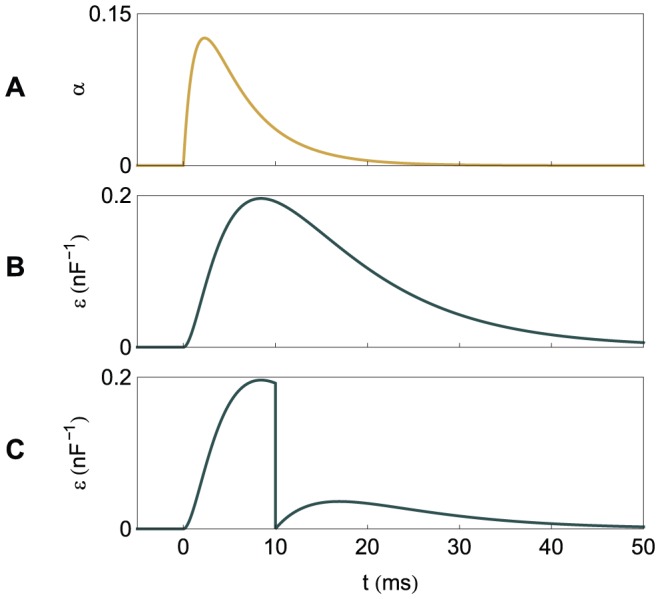
The kernels used in the simulation of the integrate-and-fire neuron. (A) The 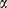 kernel. (B), (C) The 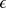 kernel. In (B) there is no postsynaptic spike. In (C), a postsynaptic spike is fired at 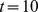 ms. A presynaptic spike is received at 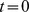.

In order to be able to test the learning rules in a computer simulation, we must define the forms of the 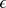 and 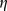 kernels of the Spike Response Model. We define them to correspond to the classical leaky integrate-and-fire neural model, which is a particular case of the Spike Response Model [21]. A further choice must be made for the form of the synaptic currents. We modeled the kernel 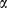 that reflects the form of the synaptic current generated by the arrival of a presynaptic spike through the synapse 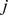 at the timing 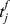 as a difference of two exponentials (double-exponential current):
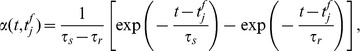
(30)for 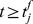, where 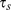 and 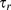 are positive parameters (time constants). The 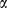 kernel is illustrated in Fig. 19 A.

The synaptic current contributed by one spike at 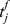 is the product of the synaptic efficacy 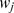 and of the normalized 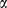 kernel:
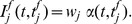
(31)The 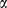 kernel is normalized:
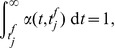
(32)and therefore the synaptic efficacy 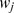 represents the total charge transmitted to the postsynaptic neuron as a consequence of one presynaptic spike.

The synaptic current generated through the synapse 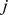 by one or more presynaptic spikes is
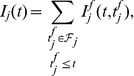
(33)and the total synaptic current received by the neuron from all synapses is
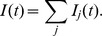
(34)


The dynamics of the membrane potential 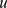 of the leaky integrate-and-fire neuron is defined by
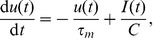
(35)where 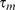 is the time constant of the neuron's leakage and 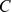 is the capacity of the neuron's membrane (we use here a scale for the membrane potential where the resting potential is 0). When the membrane potential reaches the threshold 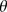, the neuron fires a spike and the membrane potential is reset to the reset potential 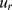.

By integrating the last equation between the moment 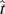 of the last emitted spike before 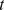, and, respectively, 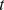, we get
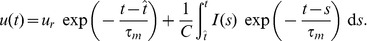
(36)By expanding 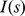 into its components generated by each presynaptic spike, we get
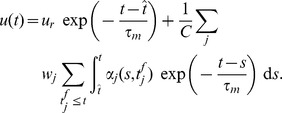
(37)We define:
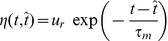
(38)

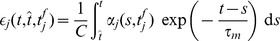
(39)We can then express the dynamics of the integrate-and-fire neuron in the form of the Spike Response Model, Eq. 6:
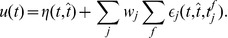
(40)


After performing the integration in Eq. 39 by taking into account the form of the 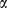 kernel given by Eq. 30, we get:
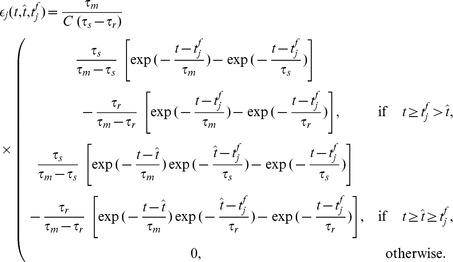
(41)The form of 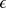 is illustrated in Fig. 19 B, C.

### Computer simulations

The learning rules were tested and explored in computer simulations. We trained an integrate-and-fire neuron with double-exponential synaptic currents, as previously described. The neuron had a membrane time constant 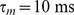, a capacity 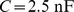, a firing threshold 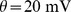, and the resting potential was 0. Except in Fig. 16, the neuron had a reset potential equal to the resting potential, 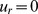. The time constants that define the dynamics of the synaptic currents were 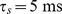 and 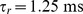.

Since we were interested in the coding of information in the precise timing of the spikes, we used an event-driven simulation [117] where the timing of input spikes were represented with machine precision and the timing of the trained neuron's spikes were computed with a precision of 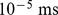.

The neuron received inputs through 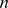 synapses. In Figs. 6, 7, 10, 11, 12, 13, 14, 15, 16, and 18 we used 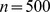, while in Figs. 9 and 17 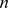 was variable, but at least 500. At the beginning of learning experiments, synaptic efficacies were generated randomly, with an uniform distribution between 0 and 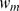. In Figs. 6 and 7 we used 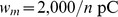, while in Figs. 9 and 10, 11, 12, 13, 14, 15, 16, 17, and 18 we used 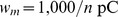.

The neuron was trained to learn 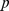 input patterns by firing a pre-determined output spike train for each of the inputs. An input pattern consisted of the ensemble of the 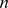 input spike trains coming through the 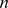 synapses during the interval 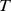. Except in Fig. 15, the length 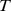 of the input patterns was of 200 ms. During learning, the input patterns were presented sequentially, in batches consisting of the 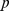 patterns. Except in Fig. 18, the synaptic changes defined by the learning rules were accumulated and were applied to the synapses at the end of each batch. A presentation of one input pattern and the simulation of the output of the trained neuron corresponding to this input is called a trial. Each batch of presentations of the 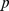 patterns (trials) is called an epoch. Except in Fig. 14, at the beginning of each trial, the membrane potential of the neuron was reset to 0.8 of the value of the firing threshold 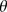; this value was used in order to allow the neuron to fire even at moments close to the beginning of the trial.

The learning rates that we used were, for E-learning: 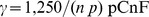 in Fig. 6, 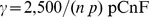 in Fig. 7, and 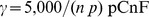 in Figs. 9, 10, 11, 14, 15, 16, and 18. We also used 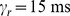. For I-learning, the learning rates were 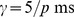 in Figs. 6 and 7, and 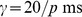 in Figs. 9 and 18. These values were close to the optimal ones. The inverse proportionality to 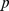 reflects the accumulation of the synaptic changes during the presentation of the 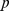 patterns. The inverse proportionality with 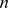 for E-learning but not for I-learning reflects that the average value of the synaptic efficacies scales inversely proportional to the number of synapses, for about the same behavior of the neuron. In I-learning the changes of synaptic efficacies are proportional to the synaptic current, which is already scaled inversely proportional to the number of synapses as it is proportional to the synaptic efficacy, and thus no scaling with 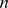 is needed for the learning rate. We also used 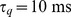.

The I-learning rule implies that changes of synaptic efficacies are proportional to the synaptic current and thus to the values of the synaptic efficacies. Thus, if the initial synaptic efficacies are all positive, they cannot become negative if the learning rates are sufficiently small. The application in batches of synaptic changes or rounding errors may, however, allow a sign change of the synaptic efficacies in a computer simulation. In our simulations with I-learning we enforced that synaptic efficacies stayed positive, by using a hard bound. For E-learning, we allowed the synapses to switch sign, according to the changes suggested by the learning rule.

In Figs. 6, 7, 8, 9, 10, 11, 14, 15, 16, 17, and 18, the input spike trains consisted, for each of the 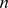 synapses, of one spike generated at a random timing, distributed uniformly between 0 and 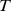 (latency coding of information).

In Fig. 7, we considered that the actual spike matched the target one if there was exactly one actual spike and its timing was within 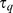 of the target timing. The probability 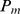 that the fired spikes matched the target ones was the number of patterns within a trial for which the actual spikes matched the target one, divided by the total number of patterns, 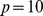.

In the experiments presented in Figs. 7, 8, 9, 10, 11, 12, 13, 14, 15, 16, 17, and 18, we trained the neuron to perform classifications by setting its target output to be the same for several different, randomly generated, inputs. The number 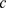 of the different outputs was the number of categories into which the neuron classified the 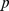 input patterns. We assigned equal number of patterns into each category, and therefore 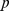 was an integer multiple of 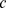. We considered that an input-output association was learned correctly by the trained neuron if the number of the actual output spikes was the one in the target spike train and each of the output spikes was fired within less than 1 ms of the target timing. We considered that the chronotron was able to learn correctly a particular setup if all input-output mappings were learned correctly in no more than 10,000 epochs.

The output used a latency-coded representation of the information. Except in Figs. 11 and 14, the target spike train for each category 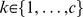 consisted of one spike at 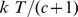.

For each realization of the experiments, both the input patterns and the initial synaptic efficacies were generated randomly. In Figs. 9, 10 and 12, for various values of the number of inputs 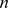 we increased the load 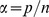 until the chronotron was not able to learn correctly all the 500 random realizations of the setup. The capacity for a particular setup was the maximum load for which the chronotron was able to learn correctly that setup, lower than the first load for which the chronotron was not able to learn correctly the setup. In Figs. 11, 13, 14, 15, 16, and 18, the capacity was the maximum load for which chronotron was able to learn correctly a particular setup, lower than the first load for which the chronotron was not able to learn correctly more than 1% of the 500 random realizations of the setup. For each setup and load, we recorded the minimum number of epochs 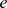 after which the chronotron was able to learn correctly the setup.

In Figs. 9, 12, 13, 15, 16, and 18, the experiments were performed with 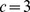 categories, in Figs. 7, 11 and 14 with 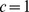, in Fig. 17 with 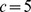, and in Fig. 10 the number of categories varied.

For E-learning, simulations for 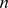 higher than 2,000 were not performed in Fig. 9 because of the high computational cost, due to the high capacity resulted through this learning rule. For example, the simulations required for obtaining the results presented for 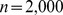 took about 13 days on a computer with 8 Xeon cores running in parallel at 2.33 GHz.

For the simulations using ReSuMe in Fig. 9, we used 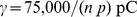 and 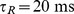. These were optimal parameters, that led to the lowest occurrence of cases where correct learning was not achieved for 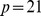, 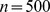 (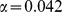), 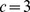, from a scan of the 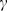, 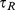 parameter space with a resolution of 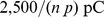 and, respectively, 2 ms. We also used 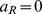. We verified that, for 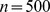, the capacity did not increase if we used nonzero 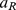, for various values spanning several orders of magnitude.

In Fig. 1, we used 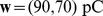. In Fig. 2, learning converged for 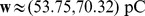. In Figs. 1, 2, and 4, we used 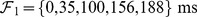 and 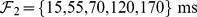. In Figs. 2 and 4, the target spike train was 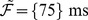.

In Fig. 3, we used 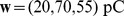, 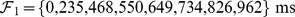, 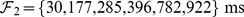, and 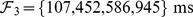.

### Exploring the dependence on the setup parameters

In Fig. 11, we explored the chronotron performance as a function of the number of output spikes per trial, for a setup where the chronotron had to fire the same output for all inputs. The setup was as in Fig. 9, with E-learning and 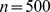, except that 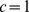, and that the output consisted of 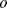 output spikes, placed at 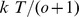, for 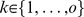.

**Figure 20:**
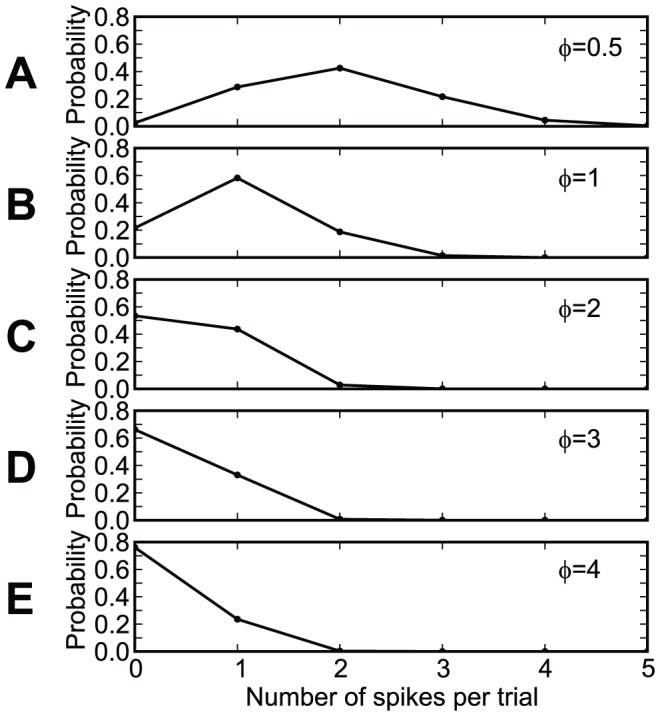
The distribution of the number of input spikes per trial, for inputs generated using a Gamma process, as in **Fig. 12**. (A) The normalized average period is 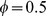. (B) 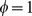. (C) 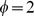. (D) 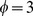. (E) 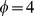.

In Fig. 12, we explored the chronotron performance as a function of the firing rate of the inputs. Here, the input spike trains were generated using a Gamma process of order 3 and time constant 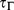, i.e. the interspike intervals were generated randomly with a probability distribution, for an interspike interval 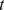,
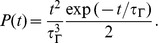
(42)This leads to input spike trains having an average firing rate of 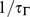 and an average interspike interval (period) 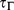. The learning rate was adapted to the input firing rate, 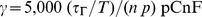. Except the input and the learning rate, the setup was as in Fig. 9, with E-learning and 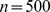. We studied the performance as a function of the normalized average period 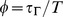. The probability distribution of the number of input spikes per trial, for several values of 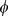, is illustrated in Fig. 20.

In Fig. 13, at the beginning of the experiment, each input spike train was set up as either one spike generated at a random timing, distributed uniformly between 0 and 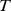, as before, or, with a probability 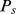, of no spikes. Input patterns did not change during learning. We explored the chronotron performance as a function of the no firing probability 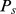. The learning rate was adapted as 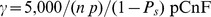. Except the input and the learning rate, the setup was as in Fig. 9, with E-learning and 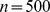.

In Fig. 14, we explored the chronotron performance as a function of the timing of the output spike and of the initial state of the membrane potential. The neuron had to learn to have the same output for all inputs. The output was one spike at a given timing 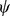 relative to the beginning of the trial. At the beginning of each trial, the membrane potential 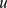 was either set to 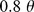, as in the other experiments (stable initial state), or was generated randomly, with a uniform distribution, between 0 and 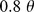 (random initial state). Except the target output and the initial state of the membrane potential, the setup was as in Fig. 9, with E-learning, 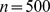, and 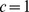.

In Fig. 15, we explored the chronotron performance as a function of the trial length 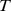. Except the trial length, the setup was as in Fig. 9, with E-learning and 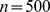. Since the setup is invariant to a change of the time scale, the relevant parameters are the relative time scales 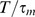, 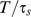, 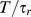, 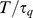.

In Fig. 16, we explored the chronotron performance as a function of the reset potential 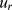. Except the reset potential, the setup was as in Fig. 9, with E-learning and 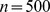.

In Fig. 17, we used parameters optimized for fast learning for a setup with a relatively low load, 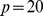, 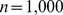 (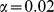), with 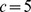. Parameters were optimized to lead to the minimum average number of learning epochs needed for correct learning for this setup. Averages were computed over 500 realizations with random initial conditions and inputs. Inputs and outputs were latency-coded, as in Fig. 9. The parameters that resulted from the optimization were: for E-learning, 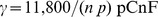, 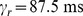, 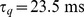; for I-learning, 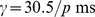; for ReSuMe, 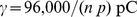, 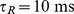, 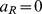.

In Fig. 18, we explored the dependence of chronotron performance on when synapses are updated during the simulation. Synapses were updated by applying the synaptic changes defined by the learning rules and accumulated between the updates. Here we updated the synapses either at the end of each batch (epoch) consisting of presentations of the 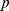 input patterns (batch updating), as in the other experiments; at the end of each trial (presentation of one input pattern) (trial updating); or, for I-learning, immediately following each actual or target postsynaptic spikes, as in Fig. 5 (online updating). In trial updating, the order of the presentations of the input patterns was chosen randomly at the beginning of each batch. Except the method for updating synapses, the setup was as in Fig. 9, with E-learning and 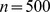.

### The information capacity of the chronotron

The load 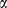 of a neuronal classifier is the number of patterns it memorizes per each input synapse of the neuron. If the neuron has 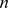 input synapses and it memorizes 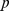 patterns, the load is
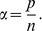
(43)


We define the information load 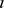 of a neuronal classifier as the quantity of information it can store for each of the input patterns for which it memorizes the correct output, per each input synapse of the neuron. We assume that the patterns are classified into 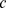 categories, and the same number of patterns is assigned to each category. The neuron stores then 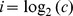 bits of information for each pattern, and the information load is
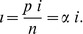
(44)


The information capacity 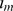 of a neuronal classifier is the maximum information load it can carry. It depends on both the maximum load 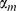 it can carry as well as on the maximum quantity of information it can store for each pattern, if they are independent.

For the perceptron and the tempotron, which can classify patterns in just 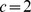 categories, we have 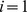 and thus the information load equals the (pattern) load and the information capacity equals the (pattern) capacity.

For chronotrons with latency coding of their outputs, firing one spike per trial, the information capacity depends on the temporal precision of the output spike and on the duration of the interval in which the output spike can be fired with no loss of capacity. We consider that 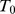 is the time interval, at the beginning of each trial, where, if the target spike is located, learning capacity is reduced (see Fig. 14). A chronotron firing one output spike per trial, having a precision of the output spike of 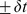, can encode, for a trial duration 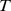, at most 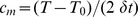 categories. The information capacity of the chronotron is then
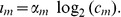
(45)The maximum capacity obtained in our simulations for E-learning was 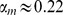, and simulations showed that this does not depend on the number of categories 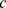 (Fig. 10). For 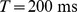, 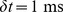, 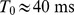, we get the maximum number of categories 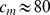, the corresponding information memorized per pattern 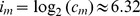 bits per pattern, and the information capacity 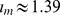 bits per input synapse.

An even higher capacity can be obtained if we also consider output spike trains consisting of more than one spike per trial.

Barak and Tsodyks [57] have developed a learning rule that allows an integrate-and-fire neuron with exponential currents to recognize input patterns from a given set, by increasing its firing rate for learned patterns in comparison to the one for background inputs. The maximum number of patterns that this rule can learn is 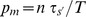, where 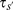 is the decay time constant of the exponential neurons. Thus, the capacity of this rule is 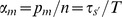. If we extrapolate this result to neurons with double-exponential currents by assuming that the same relationship applies if we consider the largest time constant of the double-exponential current, 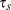 in our case, instead of 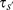, then the capacity of a neuron for recognizing patterns would be, for our setup, 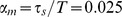. It can be seen then that the capacity that we obtained in simulations through E-learning, about 0.22, for having a particular, precisely-timed spike output pattern for each input, is about an order of magnitude larger than the capacity computed for just the recognition of patterns using a firing rate code.

### The algorithm for computing the sets of spikes to be removed, inserted or moved

Victor and Purpura [25], [115] presented an algorithm for computing the distance between spike trains that they defined, but not one for indicating the pairs of matching spikes (consisting of one spike from each spike train) and the sets of independent spikes that the distance implies. This information represents the structure of the pair of spike trains, as defined by the metric. Here we extend the Victor & Purpura algorithm with the capacity of computing this structure.

When the two spike trains that are compared consist of one that is fixed (the target one) and one that is modifiable (the actual one), as in our supervised learning problem, the set 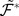 of independent spikes in the target spike train corresponds to timings when new spikes should be created in the actual spike train; the set 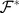 of independent spikes in the actual spike train represents the spikes that have to be removed; and pairs of matching spikes define the set of actual spikes that have to move and their targets.

The original algorithm [25], [115] computes the distance between spike trains inductively, as follows. Let 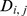 be the distance between the spike trains composed of the first 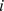 spikes of 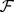, 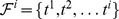, and, respectively, the first 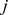 spikes of 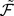, 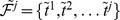. 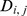 is computed as:
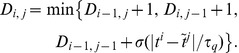
(46)The elements from which the induction starts are 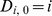 and 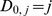, because it is considered that 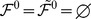 (the distance between a train of 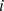 spikes and a train of no spikes is 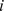 because all spikes must be removed or correspondents for all must be inserted for a cost of 1 each). If there are 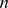 spikes in 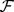 and 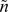 spikes in 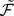, the algorithm needs to use a 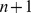 by 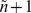 matrix that stores the 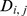 values for the various 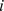 and 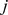. The actual distance between the full spike trains is 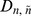, the element at the bottom right of the matrix. Because the computation of each 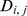 element requires all the three values placed above, left and above left in the matrix, all the elements in the matrix have to be computed inductively.

The choice of the minimum of the three values performed at the computation of each element 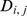 of the matrix (except the ones in the left and top edges of the matrix, 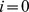 or 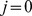) reflects an optimal choice of the status of the last spikes in the partial spike trains corresponding to the considered element. The optimal status of the last spikes depends on the structure of the pair of partial spike trains that precedes them. If the minimum is 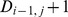, then the spike at 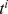 has a contribution of 1 to the distance and it is thus independent of any spike in the reciprocal spike train 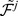; the spike at 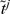 may or may not be independent, as a function of the structure of the 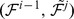 pair of spike trains. If the minimum is 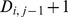, then the spike at 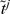 is independent of any spike in the reciprocal spike train 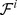; again, the spike at 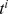 may or may not be independent, as a function of the structure of the 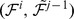. If the minimum is 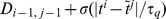 then the actual spike at 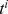 is linked to the target one at 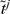 and will have to move towards it.

If more than one of the three values have the minimum value, then, at least theoretically, they might represent different, alternative choices of the optimal structure of the 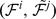 pair of spike trains. We will consider here that a pair of spikes 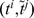 is linked if and only if 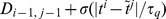 is a strict minimum, i.e. it is the only one of the three choices that corresponds to the minimum value of 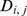. If it is equal to another minimum, the link has just been broken and we will consider the alternative structure. If 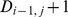 and 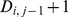 are equal minima, they might correspond to different structures, as a function of the structures of 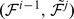 and 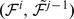. However, if these structures involve pairs of linked spikes, it is extremely improbable that the equality 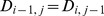 will hold exactly, especially in a numerical computer simulation. The equality can hold with a non-vanishing probability when all spikes in 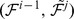 and 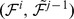 are independent, in which case the two alternative structures for 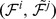 are actually identical, since they both consider that all the spikes are independent. Even in the improbable case that the equality holds when links do exist, for our purpose of supervised learning is is sufficient to consider only one of the alternatives, as long as we are consistent in the choice of this alternative.

It can be shown that, if 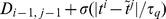 is a strict minimum value for computing 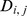, then 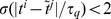 and thus the two spikes are linked (not independent), as follows. The addition of a spike at 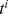 to the pair of spike trains 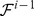 and 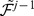 can increase the distance with at most 1, because in the worst case the spike will be removed for a cost of 1. We thus have
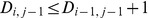
(47)

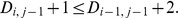
(48)But if 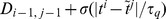 is a strict minimum, then
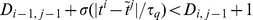
(49)and from the last two equations we get
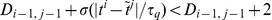
(50)

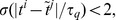
(51)which was to be demonstrated.

The algorithm for computing the structure of the pair of spike trains 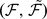 has to compute the structure inductively, along with the computation of the distance between the spike trains. We will thus have to store the structure of all pairs of partial spike trains 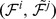 for 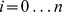 and 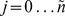. This structure is defined by indicating for each spike whether it is independent or not; if it is linked (not independent), it will also have to indicate the index of the spike in the other train to which it is linked. The structure of 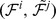 is formed by the pair 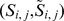 where the first element is the structure information for 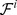 when used for computed 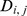 and the second element is the structure information for 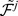 when used for computed 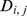. More precisely, 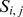 is a list in which each element indicates whether the corresponding spike 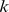, with 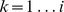, is independent, which we denote through an element 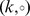; or whether the spike is linked to a spike 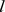 in the other spike train, which we denote through an element 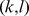. The list 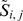 has an analogous meaning for the spikes in the target spike train 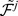.

Algorithm 1 lists the entire procedure of computing the structure of the pair of spike trains along with the distance between them.

**Input:** The pair of spike trains 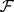, 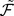; the parameter 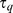; the function 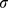


**Output:** The distance between the spike trains and the structure of the spike trains corresponding to this distance


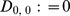
;


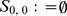
;


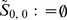
;

*Set the left edge of the matrix*

**for** 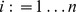 **do**


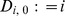
;


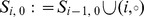
;


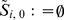


*Set the top edge of the matrix*

**for** 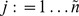 **do**


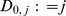
;


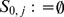
;


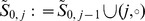
;

*Perform the inductive computation*

**for** 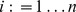 **do**

**for** 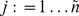 **do**

   Compute 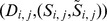



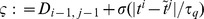
;

**if** 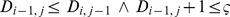 **then**

    Spike 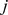 is independent


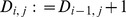
;


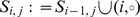
;


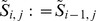
;

**else if** 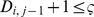 **then**

    Spike 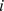 is independent


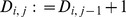
;


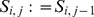
;


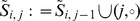
;

**else**

    Spikes 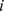 and 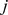 are linked


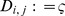
;


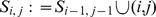
;


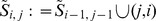
;

**return** 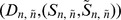;

**Algorithm 1:** The algorithm for computing the distance between two spike trains and the structure of these spike trains corresponding to the distance. The text in italics represents comments.

### Average displacement for gaussian jitter

In Fig. 7, input spikes were displaced randomly around the reference timing according to a gaussian distribution with an amplitude 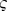. The probability density of a (positive or negative) displacement 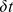 is
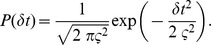
(52)The probability density of a given displacement, in absolute value, is
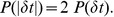
(53)The average displacement (in absolute value) is then
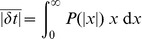
(54)

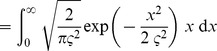
(55)

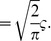
(56)For 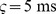, we get 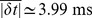.
